# A validated LC–MS/MS method for clinical pharmacokinetics and presumptive phase II metabolic pathways following oral administration of *Andrographis paniculata* extract

**DOI:** 10.1038/s41598-023-28612-1

**Published:** 2023-02-13

**Authors:** Phanit Songvut, Nanthanit Pholphana, Tawit Suriyo, Nuchanart Rangkadilok, Duangchit Panomvana, Porranee Puranajoti, Jutamaad Satayavivad

**Affiliations:** 1 Laboratory of Pharmacology, Chulabhorn Research Institute, 54 Kamphaeng Phet 6 Rd., Laksi, Bangkok, 10210 Thailand; 2 Center of Excellence on Environmental Health and Toxicology (EHT), OPS, MHESI, Bangkok, 10400 Thailand; 3 Translational Research Unit, Chulabhorn Research Institute, Bangkok, 10210 Thailand

**Keywords:** Drug development, Pharmacokinetics, Mass spectrometry

## Abstract

*Andrographis paniculata*, a medicinal plant in Thailand national list of essential medicines, has been proposed for treatment of patients with mild to moderate coronavirus disease 2019. This study aims to develop a highly selective and sensitive liquid chromatography triple quadrupole tandem mass spectrometry method for quantitative determination of major diterpenoids in plasma and urine with application in pharmacokinetics. Chromatographic separation was performed on C18 column using a gradient mobile phase of water and acetonitrile. Mass spectrometry was analyzed using multiple reaction monitoring with negative ionization mode. This validated analytical method was very sensitive, less time consuming in analysis, and allowed the reliability and reproducibility on its application. The clinical pharmacokinetics was evaluated after single oral administration of *A. paniculata* extract (calculated as 60 mg of andrographolide). The disposition kinetics demonstrated that major diterpenoids could enter into systemic circulation, but they are mostly biotransformed (phase II) into conjugated glucuronide and sulfate metabolites. These metabolites are predominantly found in plasma and then extremely eliminated, in part through urinary excretion. The successful application of this analytical method supports its suitable uses in further clinical benefits after oral administration of *A. paniculata.*

## Introduction

Coronavirus disease 2019 (COVID-19), caused by the severe acute respiratory syndrome coronavirus 2 (SARS-CoV-2), is a global health crisis that is driving efforts to identify alternative medicinal plants to be used in COVID-19 treatment. There is evidence that the Acanthaceae family member *Andrographis paniculata* (Burm.f.) Nees, can alleviate the severity of symptoms and shorten the duration of treatment in patients with mild to moderate COVID-19^1,2^. The recommended dose of *A. paniculata* extract for relief of symptoms in uncomplicated upper respiratory tract infections (URTIs) is 20 mg orally administered three times a day (calculated as 60 mg/day of andrographolide)^3–5^. However, a higher dose of the extract, 60 mg/dose of andrographolide three times a day (180 mg/day of andrographolide), has been used in a pilot study of patients who tested positive for COVID-19 in Thailand^2^. While the clinical benefits of the extract (60 mg of andrographolide/dose) have been evaluated, the data on the pharmacokinetics of this dose remain limited. Therefore, one of the objectives of this study was to investigate the clinical pharmacokinetics of andrographolide (AP1), a major phytoconstituent of this plant, and of other diterpenoid derivatives^6–8^ including: 14-deoxy-11, 12-didehydroandrographolide (AP3), neoandrographolide (AP4), and 14-deoxyandrographolide (AP6) after a single oral administration of *A. paniculata* extract (equivalent to 60 mg/dose of andrographolide).

Liquid chromatography-tandem mass spectrometry (LC–MS/MS) is a powerful technique widely used in biological analysis, including in the screening of lead candidates, determination of metabolites, and pharmacokinetic investigation^9^. Additionally, the advanced liquid chromatography-quadrupole time-of-flight mass spectrometry (LC-QTOF/MS) technique was established for compound screening and the metabolic pathways of all related metabolites were proposed in this study. Multiple reaction monitoring (MRM) is a targeted highly specific and sensitive MS technique^10^. The MRM selection coupled with triple quadrupole MS/MS (LC-QqQ-MS/MS) enables the selective quantification of specific compounds in complex mixtures. Literature reviews considering the existing quantitative determination of active compounds of *A. paniculata* extract in biological samples illustrated that most of the analytical inquiry has focused on andrographolide^11–13^, with only one study providing the determination of four major diterpenoids in plasma^6^. The previous method, however, limits the quantifiable detection of andrographolide with LLOQ at 2.50 ng/mL^6^. Due to this limitation, there were several restricted undetectable plasma levels of diterpenoid derivatives during the elimination phase^6,7^. As indicated in the previous reports^12,14^, the concentrations of major diterpenoids in human blood circulation were observed to be very low due to their poor oral bioavailability. Therefore, andrographolide and its derivatives were less than a quantifiable limit when blood sampling was carried out over 8 h until 24 h^6^. In case of this limitation, the desired pharmacokinetic parameters cannot be obtained during this elimination period. To support the complete pharmacokinetic investigation, the present study has further developed and fully validated a rapid, highly selective and sensitive liquid chromatography–tandem mass spectrometry (LC–MS/MS) method with the lower limit of quantification < 1.00 ng/mL, that simultaneously determined the four major diterpenoids in plasma samples. Their conjugated metabolites were also determined by using enzymatic digestion.

Taken together, the previous method was applicable for determining the four major diterpenoids (AP1, AP3, AP4, and AP6) in only plasma samples^6^. For this reason, the application of present newly developed analytical method was then expanded to urine samples. The method was validated for plasma and urine samples in terms of selectivity, specificity, accuracy, precision, linearity, range, limit of quantification, recovery, matrix effects, stability tests, and hemolysis assessment, according to the acceptance criteria of the US FDA Bioanalytical Method Validation Guideline^15^ and the International Conference on Harmonization ICH Guideline M10 on Bioanalytical Method Validation^16^.

The present study is the first investigation of oral dosing of a high dose administration of *A. paniculata* extract (60 mg of andrographolide/dose) in which the pharmacokinetics of the parent compounds and their conjugated glucuronide and sulfate metabolites with the presumptive phase II metabolic pathways pathways have been simultaneously determined in both plasma and urine samples.

## Materials and methods

### Materials

Standards of andrographolide, AP1 (purity = 100.00%); 14-deoxy-11, 12-didehydroandrographolide, AP3 (purity = 99.80%); neoandrographolide, AP4 (purity = 99.67%); and 14-deoxyandrographolide, AP6 (purity = 100.00%) were supplied by Phytolab GmbH & Co.KG (Vestenbergsgreuth, Germany). The internal standard (IS) of digoxin (purity = 96.6%) was purchased from Sigma-Aldrich (St. Louis, MO, USA). The tested product, *A. paniculata* aqueous extract capsules (101.3% of the label amount of andrographolide, Lot number 119010921) was manufactured by Panaosod Co., Ltd. (Chon Buri, Thailand) in accordance with the quality standards of Good Manufacturing Practice (GMP). The assay contents of capsule composition were determined using a validated HPLC–DAD method^17^, and calculated as 20.26, 6.62, 5.28, 3.02 mg/capsule for AP1, AP3, AP4, and AP6, respectively.

For enzymatic digestion, *β*-glucuronidase type IX-A (lyophilized powder) from *Escherichia coli*, glucuronidase activity of 1,000,000–5,000,000 units/g protein (glucuronidase activity = 2,354,185 units/g protein, retested date: 04/02/2020), and sulfatase type H-1 from *Helix pomatia*, sulfatase activity ≥ 10,000 units/g solid (sulfatase activity = 16,134 units/g solid and *β*-glucuronidase activity = 353,820 units/g solid, quality release date: 25/05/2021) were obtained from Sigma-Aldrich (St. Louis, MO). For preparation of phosphate and acetate buffers, sodium dihydrogen phosphate (NaH_2_PO_4_), disodium hydrogen phosphate (Na_2_HPO_4_), glacial acetic acid (CH_3_COOH), and sodium acetate (CH_3_COONa), were obtained from Sigma-Aldrich (St. Louis, MO). Acetonitrile and methanol (HPLC grade) were purchased from Merck (Darmstadt, FR, Germany). Milli-Q water (Millipore, Bedford, MA, USA) was used in the LC–MS/MS system throughout the analysis procedures.

### Methods

#### Screening and identification of the analyte and metabolite profiling using LC-QTOF/MS analysis

For qualitative determination of untargeted compounds, liquid chromatography-quadrupole time-of-flight mass spectrometry (LC-QTOF/MS) was performed on an Agilent 6540 QTOF/MS (Agilent Technologies, USA) equipped with an Agilent 1260 infinity liquid chromatography system. The stationary phase of the chromatographic separation was performed on a 150 mm × 4.6 mm, 5.0 µm Phenomenex Luna C18 column (Phenomenex, USA) using a mobile phase consisting of 0.1% formic acid in water pH 2.5–2.7 (A) and 100% acetonitrile (B) at a constant flow rate of 0.5 mL/min with a controlled oven temperature at 35 °C, (and 10 µL) of injection volume. The elution gradient was started with 30%B, and was increased to 90%B within 20 min, then maintained at 95%B during 25–30 min followed by a post-run for 5 min.

Mass spectrometric analysis was performed using an ESI negative and positive modes with varying collision energies (10, 20, and 40 V) over a mass range of m/z 100–1200 Da. The MS conditions were as follows: capillary voltage = 3500 V; flow rate of drying gas (N_2_) = 10.0 L/min, 350 °C; nebulizer pressure = 30 psi, fragmentation voltage = 250 V for negative mode and 100 V for positive mode. All acquisition data were analyzed using MassHunter Software B.07.01 and MassHunter Qualitative Analysis Software B.08.00 (Agilent Technologies, USA). To support the metabolite identification, the MS chromatograms were determined by comparing the fragmentation patterns of metabolites and library searches in Mass Hunter, and Human Metabolome Database (HMDB). For sample preparation, pooled urine samples at 0–4 and 4–8 h were prepared using the same extraction process described in the pharmacokinetic study.

#### Development of analytical and instrument conditions for quantitative determination of four major diterpenoids using LC-QqQ-MS/MS analysis

##### LC and MS condition optimization

Liquid chromatography-tandem mass spectrometry (LC–MS/MS) was performed using a Nexera X2 LCMS-8060NX triple quadrupole mass spectrometer (Shimadzu, Japan) equipped with a SIL-40C XR autosampler, CTO-40C column oven, CBM-40lite system controller, FCV-20AH2 switching valve, DGU-403 degasser, and LC-40D XR solvent delivery (pump unit). The LC separation was conducted on a VertiSep AQS C18 column (100 mm × 3.0 mm, 3.0 µm) using a gradient mobile phase. The different solvents (including acetonitrile, methanol, and water, with or without formic acid) were tested to optimize sample elution systems.

Mass spectrometry (MS) and ionization was optimized and operated in Multiple Reaction Monitoring (MRM) mode. MS detection was fully scanned by directly injecting each standard (10.00–100.00 ng/mL) into the MS instrument without separation on a column. MS optimization was performed under the positive and negative conditions of electrospray ionization (ESI) mode. The MRM transitions and the fragmentations of parent and daughter ions for each analyte, with the corresponding collision energy, are shown in Table 1.

**Table 1:** MRM transitions and fragmentations of parent ions and daughter ions of andrographolide (AP1); 14-deoxy-11, 12-didehydroandrographolide (AP3); neoandrographolide (AP4); 14-deoxyandrographolide (AP6); and digoxin (IS).

Compounds	Precursor ion (*m/z*)	Product ion (*m/z*)	Collision Energy (volts)
Andrographolide	349.1	287.2*	15.0
	349.1	331.1	11.0
14-Deoxy-11,12 didehydroandrographolide	331.1	239.2*	21.0
	331.1	108.0	30.0
Neoandrographolide	479.2	161.0*	16.0
	479.2	317.3	23.0
14-Deoxyandrographolide	333.1	285.2*	18.0
	333.1	305.1	22.0
Digoxin (IS)	779.3	649.2*	33.6

*Quantitative ion.

#### Preparation of stock standards and working solutions

The primary stock solution (1.00 mg/mL) of the four standard diterpenoids (AP1, AP3, AP4, and AP6) and an internal standard (digoxin) were prepared and then stored at − 20 °C. Briefly, each standard was accurately weighed and dissolved in the appropriate volume of methanol (HPLC grade). Working solutions containing the four diterpenoids were then prepared from the stock solutions by serial dilution with methanol to obtain the appropriate concentrations of these four analytes. For the internal standard (IS), a further dilution of the stock solution was prepared at a concentration of 50.00 ng/mL.

#### Preparation of calibration standards and quality control (QC) samples

A calibration curve was generated from different standard concentrations that were prepared by spiking a small volume of the working solution (not more than 5% of the total volume) into blank human plasma or urine to obtain twelve final concentrations ranging between 0.98 and 1000.00 ng/mL for each analyte. Quality Control (QC) samples were also prepared by using the same method as the calibration standards; with concentrations of 2.50, 500.00, and 900.00 ng/mL for low (LQC), medium (MQC), and high (HQC) concentrations, respectively. These spiked plasma samples (calibration standards and QC samples) were then similarly extracted following the sample preparation protocol with the addition of 50.00 ng/mL of IS.

#### Plasma and urine samples extraction

An aliquot of 50 µL of plasma or urine was placed in a 1.5 mL of polypropylene centrifuge micro-tube. Protein was removed from the sample by adding 200 µL of methanol (containing 50.00 ng/mL of IS). The mixture was vortexed for 10 min and was then centrifuged at 12,000 rpm, 4 °C for 10 min. The supernatant was filtered through a 0.2-µM PVDF membrane (Chrom Tech) and transferred into a glass insert in a vial before LC‑MS/MS analysis.

#### Method validation

The bioanalytical method of LC-QqQ-MS/MS analysis was fully validated for (1) selectivity and specificity, (2) accuracy and precision, (3) linearity, range and lower limit of quantification, (4) recovery, (5) matrix effect, (6) stability, and (7) hemolyzed plasma effect, according to the US FDA Bioanalytical Method Validation Guideline^15^ and the International Conference on Harmonization ICH Guideline M10 on Bioanalytical Method Validation^16^.

### Key performance characteristics of method validation

#### Selectivity and specificity

The selectivity and specificity were evaluated by comparing between the retention time of the extracted blank plasma or blank urine and the retention time of each spiked standard or IS in the extracted samples. If any interferences were observed, the signal of their peak area should be less than 20% of the peak area of LLOQ, and less than 5% of the average peak area of IS in blank samples.

#### Accuracy and precision

For within-day accuracy and precision, four concentration levels at LLOQ, LQC, MQC, and HQC in five replicates, were determined in the same day. For determination of inter-day accuracy and precision, the same concentrations were extracted and analyzed on separate days (three different days) and used for three batch runs in five replicates. The accuracy was shown as a percentage, and the acceptance criteria was that each concentration should be within ± 15% for QC samples, and within ± 20% for the LLOQ. The precision of testing was expressed as percentage of the coefficient of variation (%CV), and the acceptance criteria was that each concentration should be less than 15% for QC samples and less than 20% for LLOQ.

#### Linearity, range and lower limit of quantification (LLOQ)

A twelve-point calibration curve was constructed by plotting the peak-area ratio (analyte:IS) against the concentration of the calibration standards. Determination of linearity was done using 3 calibration curves from each day (three separate days). The analysis requirement of linear regression was determined by the coefficient of determination (R^2^ ≥ 0.99). The acceptance criteria for accuracy and precision of the calibration curve should be within 85–115% and ± 15%CV of the nominal value, except for LLOQ, which should be within 80–120% and ± 20%CV.

To determine the lower limit of quantification (LLOQ), 5 samples at LLOQ were analyzed for 3 batch runs on separate days. The analytical signal of LLOQ should be at least five times higher than the signal of the blank plasma or blank urine. The acceptable limits of accuracy should be within 80–120% and %CV ≤ 20.

#### Recovery

The recovery of the four diterpenoids in plasma and urine was measured at three different QC concentrations: LQC, MQC and HQC (n = 5 for each concentration) for both pre-spiked standard of QC samples and post-spiked standard of blank plasma or urine. Extraction recoveries were calculated by comparing the peak area of pre-spiked standard in a blank matrix with the area of post-spiked standard in an extracted matrix at corresponding concentrations. To meet acceptance criteria, the recovery should be precise, accurate, and repeatable with %CV of recovery less than 15%.

#### Matrix effect

Matrix effects at three levels of QC concentrations (LQC, MQC and HQC) were determined by comparing the mean peak area of the samples prepared by spiking post-extracted samples with the analytes and IS in solution (without extraction) at corresponding concentrations. The %CV of the matrix effect should be within the acceptance criteria, which is less than 15%. The matrix factor (MF) was estimated based on the equation,

$${\text{MF }} = { }\frac{{{\text{mean }}\;{\text{response}}\;{\text{ of }}\;{\text{analyte}}\;{\text{ or}}\,{\text{ IS}}\;{\text{ in }}\;{\text{post-extracted sample}}\,}}{{{\text{mean }}\;{\text{response}}\,{\text{ of }}\;{\text{analyte}}\,{\text{ or }}\;{\text{IS}}\;{\text{ in}}\;{\text{ solution}}}}$$

The IS normalized MF was then calculated as

$${\text{IS normalized MF }} = { }\frac{{\text{MF of the analyte}}}{{\text{MF of IS}}}$$

The acceptance value of IS normalized MF should be within 0.8–1.2.

#### Stability

The stability of plasma and urine samples were evaluated at three different QC concentration levels (LQC, MQC and HQC, n = 3 for each level); the conditions were freeze–thaw stability, long-term stability, short-term stability, and post preparative stability in an autosampler. The evaluation of stability under freeze–thaw condition was carried out over five cycles of − 80 °C for at least 12 h of each cycle. Long-term stability was analyzed after storing the samples at − 80 °C. Short-term stability of each analyte, for up to 8 h during processing on the bench at room temperature, was also evaluated. For evaluation of post-preparative stability, the extracted samples were stored at ambient temperature (4 °C) in an autosampler for 24 h. The analyte was considered as a stable sample when the average percentage of accuracy of the QC sample was in the range of 85–115% and the %CV was not over 15%.

#### Hemolyzed plasma effect

To evaluate the effect of hemolysis of red blood cells on the quantification of analytes, hemolyzed plasma was prepared by adding hemolyzed whole blood (3% V/V) into blank plasma^16,18^, which was then vigorously vortexed. Thereafter, the working standard solution was added to the hemolyzed plasma at LQC and HQC samples (n = 6 replicates). The determinations for the analytes were compared between concentrations of each analyte in the blank plasma without hemolysis and the measured concentration in hemolyzed plasma. The measurement of all analytes was considered not to be interfered with hemolysis effect when the average of %accuracy was within a range of 85–115% and %CV was ≤ 15%.

#### Quantitative determination of conjugated metabolites using enzymatic digestion assay

##### Optimized enzymatic digestion

To optimize the hydrolysis process for determination of conjugated metabolites, enzymatic digestion with glucuronidase and sulfatase was performed under different incubation conditions. The hydrolysis reaction was initiated by adding 50 µL of either enzyme into a 50 µL of plasma or urine sample using different enzyme concentrations (25, 50, 100, 200, and 400 units/mL for sulfatase and 200, 500, 1000, 2000 and 4000 units/mL for *β*-glucuronidase). Then the mixture was incubated at different time points (T = 15, 30 min and 1, 2, 4 h). The reaction was terminated by adding 150 µL of cold methanol containing internal standard (50.00 ng/mL). After vortex mixing for 10 min, the extracted samples were centrifuged at 12,000 rpm, 4 °C, for 10 min. Lastly, 150 µL of supernatant was collected by filtering the mixture through a 0.2-µM PVDF membrane (Chrom Tech), and then transferred to an insert vial for LC–MS/MS analysis.

The standard calibration curves of each analyte for determining conjugated glucuronide and sulfate metabolites were carried out using the same optimized method as plasma and urine incubation. Briefly, working standard solutions were added into blank plasma and urine samples and then the process of incubation was performed before extraction with protein precipitation.

#### Method application to pharmacokinetic study

The validated method was applied in pharmacokinetic investigation of four major diterpenoids in healthy subjects after oral administration of *A. paniculata* extract.

##### Institutional review board and informed consent statement

The study protocol was approved by the Institutional Review Board of the Chulabhorn Research Institute (approval date: 28/08/2020, IRB number: 062/2563) and also registered with the Thai Clinical Trials Registry (approval date: 01/02/2021, TCTR20210201005). All clinical procedures were performed in compliance with the International Conference on Harmonization-Good Clinical Practice (GCP) under the Declaration of Helsinki. Informed consent was obtained from all subjects involved in the study.

##### Study design and eligibility criteria

The clinical pharmacokinetic study was designed as an open-labeled, with a single oral dosing, and conducted under a fasting condition. Four healthy subjects were recruited according to the following inclusion criteria: 18—55 years old with a body mass index (BMI) of 18–30 kg/m^2^. Study participants were identified as healthy based on laboratory testing and physical examinations. The participants received 3 capsules containing *A. paniculata* extract (calculated as 60 mg of andrographolide/dose) before breakfast with 240 mL of drinking water. Blood samples, in EDTA tubes, were taken after oral administration at 0, 10, 20, 30, 45 min, and 1, 1.5, 2, 4, 6, 8, 10, 12, 24 h post dose. All blood samples were then centrifuged at 4000 ± 100 rpm, 4 °C for 5 min. Plasma was pipetted and stored in cryotubes at − 80 °C until further analysis. Urine samples were collected during 0–4, 4–8, 8–12, 12–24, 24–32, 32–40 and 40–48 h after dosing and were then kept at − 80 °C before sample extraction.

#### Statistical analysis

Pharmacokinetic parameters were determined by non-compartmental analysis using PK solutions software, version 2.0 (Summit Research Services). The maximum concentration of four targeted compounds (C_max_) and the time to reach maximum concentration (T_max_) were taken directly from the individual plasma concentration versus time profiles. The area under the curve from time zero to the last sampling time (AUC_0-t_) was estimated using the linear trapezoidal rule. The apparent volume of distribution (Vd/F) and the elimination half-life (t_1/2_) were determined using non-compartmental analysis.

Statistical analysis was performed using STATA statistical software (StataCorp, USA). Continuous data were illustrated as mean ± SD, while half-life and T_max_ were expressed as median [IQR]. Graphs of the comparative pharmacokinetics results were generated using GraphPad Prism 9.3.0 (GraphPad Software, USA).

#### Data availability

The datasets generated and/or analyzed during the present study are not publicly available due to ethical concerns and confidentiality agreements but are available from the corresponding author on reasonable request that needs a consensus from colleagues.

## Results

### Screening and identification of the analyte and metabolite profiling using LC-QTOF/MS analysis

**Figure 1:**
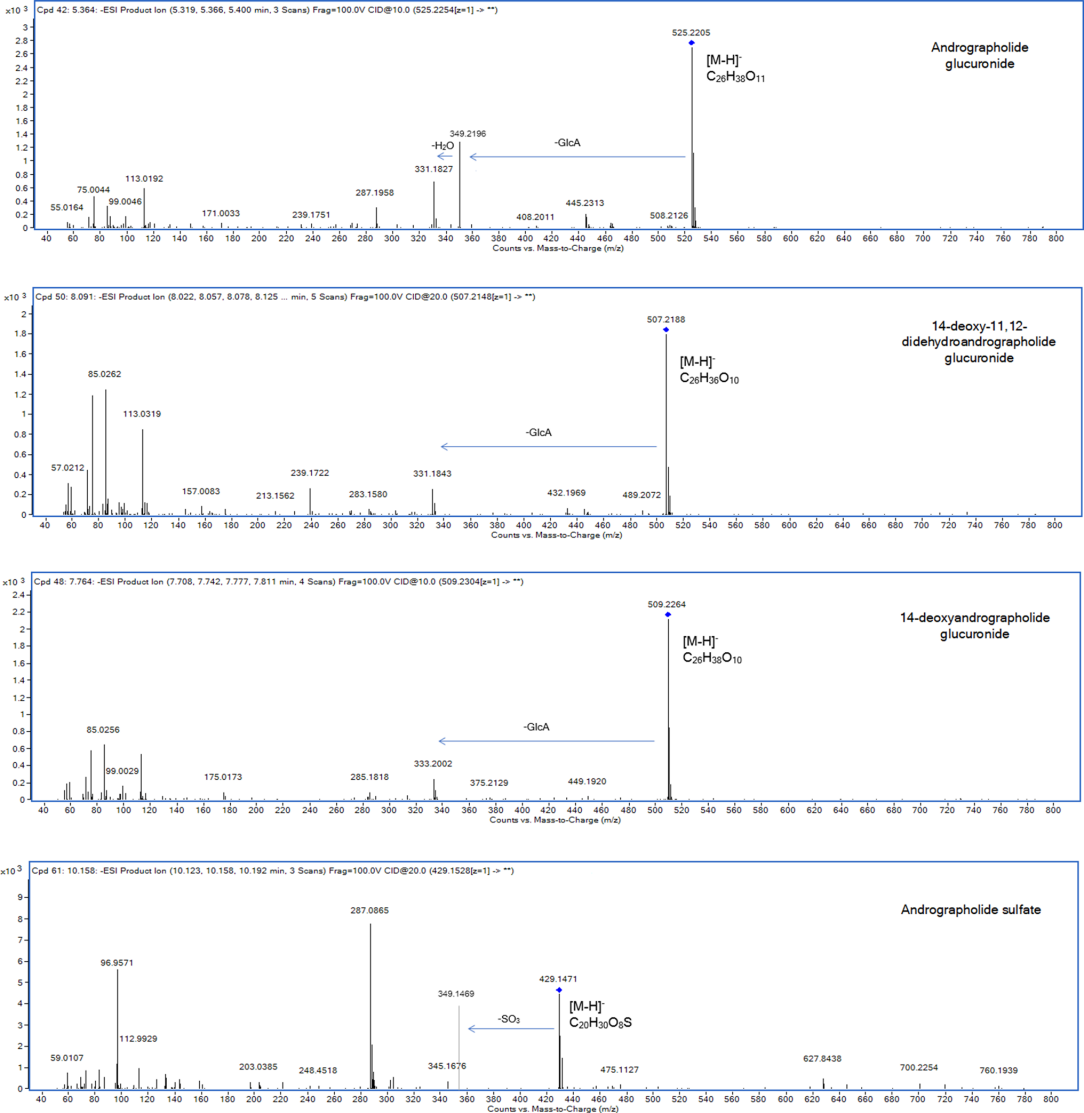
MS/MS spectra of compounds or tentative metabolites detected in human urine after a single oral administration of A*. paniculata* extract.

The compound profiling after oral administration of *A. paniculata* extract was analyzed in urine samples. A total of four compounds were tentatively identified using the chromatographic spectra from LC-QTOF/MS. The proposed metabolic pathways were associated with phase II conjugation. Three compounds (M1-3) involved in the glucuronidation pathway, and one compound (M4) related to the sulfation pathway (Table 2 and Fig. 1).

**Table 2:** Compound screening and identification after oral administration of *A. paniculata* extract.

Metabolites	Rt	Formula	Molecular ions (m/z)	Diff calculated-observed m/z (ppm)	Fragmentation (m/z)	ESI–MS mode	Identification	Metabolic Pathway
M1	5.36	C_26_H_38_O_11_	[M–H]^−^ = 525.2205	0.0136	349, 331, 287	Negative	Andrographolide glucuronide (AP1 glucuronide)	Glucuronidation
M2	8.09	C_26_H_36_O_10_	[M–H]^−^ = 507.2188	0.0048	331, 239, 108	Negative	14-Deoxy-11,12-didehydroandrographolide glucuronide (AP3 glucuronide)	Glucuronidation
M3	7.76	C_26_H_38_O_10_	[M–H]^−^ = 509.2264	0.0128	333, 305, 285	Negative	14-Deoxyandrographolide glucuronide (AP6 glucuronide)	Glucuronidation
M4	10.15	C_20_H_30_O_8_S	[M–H]^−^ = 429.1471	0.0118	349, 287, 96	Negative	Andrographolide sulfate (AP1 sulfate)	Sulfation*

*The tentative conjugated of AP3 sulfate and AP6 sulfate were omitted in this table due to the detection limit of LC-QTOF/MS technique, when low concentration of untargeted metabolite was used for analysis. To obtain the concentrated metabolites, the additional process of sample preparation needs to be considered in further studies.

#### Compound 1 (M1, t_R_ = 5.36)

The MS data detected a deprotonated ion [M–H]^−^ at m/z 525.2205 (calculated mass 525.2341) in negative ESI mode. This m/z was corresponded to the molecular formula of C_26_H_38_O_11_. The MS/MS fragmentation showed a successive loss of glucuronic acid and H_2_O (C_26_H_38_O_11_–176 Da–18 Da) at 349.2196 and 331.1827, and the product ions of M1 were consistent with the fragment pattern of andrographolide (Table 2), suggesting that M1 is a glucuronide conjugation of andrographolide (AP1 glucuronide).

#### Compound 2 (M2, t_R_ = 8.09)

The extracted-ion chromatogram (EIC) at 507.2188 (calculated m/z 507.2236) in negative ESI of a deprotonated molecule [M-H]^-^, was used to identified the molecular formula for M2 (C_26_H_36_O_10_). The eliminated glucuronide (-176 Da) of M2 exhibited a fragmented molecule at 331.1843. The specific fragmentation (507 → 331 → 239 → 108) (Table 2), corresponds to the pattern of 14-deoxy-11,12-didehydroandrographolide (AP3), indicating that M2 is a glucuronide conjugation of AP3 (AP3 glucuronide).

#### Compound 3 (M3, t_R_ = 7.76)

The MS chromatogram of M3 demonstrated the presence of the molecular structure of glucuronide. The deprotonated ion [M–H]^−^ was observed at m/z 509.2264 (calculated m/z 509.2392), which corresponded to a base formula of C_26_H_38_O_10_. The MS fragmentation indicated a deduction of glucuronide in M3 (C_26_H_38_O_10_-176 Da) at m/z 333.2002. The MS product ions were observed at 333, 305, and 285, which were determined to be a characteristic fragmentation of 14-deoxyandrographolide (AP6) (Table 2). Therefore, M3 was identified as 14-deoxyandrographolide glucuronide (AP6 glucuronide).

#### Compound 4 (M4, t_R_ = 10.15)

Compound 4 (M4) at retention time 10.15 min, showed a deprotonated molecule [M–H]^−^ at m/z 429.1471 (calculated m/z 429.1589, molecular formula C_20_H_30_O_8_S). This m/z was 80 mass units (SO_3_) higher than that of andrographolide (AP1). A subsequent fragmentation product was detected at m/z 349.1469, which was due to the neutral loss of SO_3_ molecule. The product ions were 349/287/96 (Table 2), suggesting that M4 was andrographolide sulfate (AP1 sulfate).

#### Selection of analytes for further quantitative determination

The WHO monograph on selected medicinal plants indicated the major compounds of *A. paniculata* including andrographolide, deoxyandrographolide, 11,12-didehydro-14-deoxyandrographolide, neoandrographolide, andrographiside, deoxyandrographiside, and andropanoside^8^. However, the aqueous extract of *A. paniculata* used in this clinical study composed of andrographolide (AP1), 14-deoxy-11, 12-didehydroandrographolide (AP3), neoandrographolide (AP4), and 14-deoxyandrographolide (AP6). Based on the finding of compound screening, the conjugated glucuronide and sulfate were detected in the metabolite profiling after oral administration of *A. paniculata* extract. Considering this evidence and integrating all supporting data, the four major diterpenoids (AP1, AP3, AP4, and AP6) and their respective conjugated glucuronide and sulfate metabolites have been selected as candidate analytes for determining their concentrations in plasma and urine samples, subsequently applied in this pharmacokinetic interpretation.

### Chromatographic and mass spectrometry conditions of LC-QqQ-MS/MS

#### LC optimization

Chromatographic conditions were optimized to obtain a maximum intensity, and a high sensitivity with well-quantified chromatograms of each candidate analyte (AP1, AP3, AP4 and AP6). Addition of formic acid resulted in peak distortion and a decrease in negative signal intensity of the AP1 parent compound, as described by Xu et al.^11^. Therefore, this study achieved LC separation by using water and acetonitrile, without adding formic acid in mobile phase.

Finally, optimal chromatographic separation was achieved by using a mobile phase of Milli-Q water (A) and acetonitrile (B) following elution gradients: during 0–1.5 min maintained at 40%B, then increased to 100%B during 1.5–3.0 min, and maintained at 100%B for 3.0–4.5 min. The gradient was returned to 40%B at 4.6–6.0 min. Column temperature was maintained at 40 °C during the LC separation with a constant flow rate of 0.5 mL/min, and 10 μL of injection volume.

#### Mass spectrometric conditions

The MS system, coupled with a (-)ESI ion source, was used in this analytical method due to the significant intensity observed in the negative ionization. The MS quantification was obtained by monitoring precursor/product ion transitions (m/z) at 349.1/287.2, 331.1/239.2, 479.2/161.0, 333.1/285.2 and 779.3/649.2 for AP1, AP3, AP4, AP6, and IS, respectively (Table 1, Fig. S1). Data acquisition and determination analysis were performed by using LabSolutions LCMS software (Shimadzu, Japan).

In summary, the instrument settings for MS analyses were as follows: nebulizer gas flow = 3 L/min, heating gas flow = 10 L/min, drying gas flow = 10 L/min, interface temperature = 150 °C, heat block temperature = 250 °C, and DL temperature = 200 °C.

### Method validation results

The new validated method in this study has the ability to fully determine the four major diterpenoids in both plasma and urine, using a small volume of sample (50 µL) and only requiring a short analysis time (6 min) with the lower limit of quantification < 1.00 ng/mL.

#### Selectivity and specificity

There was no interference effect from the endogenous matrix at the retention time of all analytes and IS. The representative chromatograms of blank plasma and urine; and the chromatograms with the retention times of the spiked four diterpenoid standards or IS in methanol, blank plasma and blank urine, are shown in Figs. S2, S3 and S4, respectively.

#### Accuracy and precision

The %CV for intra-day and inter-day precision of each analyte in plasma sample (Table 3a) were within the ranges of 3.14–10.29% and 0.76–6.06%, respectively, except for LLOQ, which was detected within 9.40–17.03% and 5.99–13.43%, respectively. For urine sample (Table 3b), the %CV for intra-day and inter-day precision were within the ranges of 3.54–9.37% and 0.41–4.24%, respectively; LLOQ was observed within 13.16–15.07% and 5.33–7.62%, respectively. All results of accuracy in plasma and urine samples, which are summarized in Table 3a, b, were within the acceptable limits: 89.15–104.57%, 96.48–102.41%, 91.96–104.95% and 96.61–102.39% for plasma intra-day, plasma inter-day, urine intra-day and urine inter-day, respectively. (Supporting information, Tables S1 and S2).

**Table 3:** The intra-day and inter-day accuracy and precision of four major diterpenoids: andrographolide (AP1); 14-deoxy-11, 12-didehydroandrographolide (AP3); neoandrographolide (AP4); and 14-deoxyandrographolide (AP6) in plasma (a) and in urine (b) samples.

(a) Plasma							
Spiked concentration in plasma (ng/mL)		Andrographolide (AP1)			14-Deoxy-11,12-didehydroandrographolide (AP3)		
		Measured concentration (ng/mL)	(%) Accuracy	(%) CV	Measured concentration (ng/mL)	(%) Accuracy	(%) CV
*Intra-day*							
LLOQ	0.98	0.90 ± 0.10	92.12	11.58	0.88 ± 0.13	90.11	14.20
LQC	2.50	2.50 ± 0.23	99.86	9.38	2.45 ± 0.25	98.15	10.29
MQC	500.00	468.92 ± 17.63	93.78	3.76	478.11 ± 14.99	95.62	3.14
HQC	900.00	911.23 ± 30.09	101.25	3.30	926.73 ± 37.57	102.97	4.05
*Inter-day*							
LLOQ	0.98	0.99 ± 0.08	101.42	7.97	0.97 ± 0.13	98.81	13.43
LQC	2.50	2.51 ± 0.14	100.37	5.66	2.46 ± 0.04	98.27	1.50
MQC	500.00	483.56 ± 14.37	96.71	2.97	491.63 ± 17.25	98.33	3.51
HQC	900.00	918.30 ± 12.38	102.03	1.35	907.68 ± 18.00	100.85	1.98

Spiked concentration in plasma (ng/mL)		Neoandrographolide (AP4)			14-Deoxyandrographolide (AP6)		
		Measured concentration (ng/mL)	(%) Accuracy	(%) CV	Measured concentration (ng/mL)	(%) Accuracy	(%) CV
*Intra-day*							
LLOQ	0.98	1.00 ± 0.17	101.88	17.03	0.87 ± 0.08	89.15	9.40
LQC	2.50	2.57 ± 0.21	102.88	7.98	2.29 ± 0.10	91.46	4.20
MQC	500.00	473.23 ± 19.49	94.65	4.12	472.87 ± 18.46	94.57	3.90
HQC	900.00	941.15 ± 36.25	104.57	3.85	910.69 ± 34.22	101.19	3.76
*Inter-day*							
LLOQ	0.98	0.95 ± 0.06	97.44	5.99	0.94 ± 0.11	96.48	11.35
LQC	2.50	2.56 ± 0.09	102.41	3.68	2.44 ± 0.15	97.69	6.06
MQC	500.00	488.68 ± 14.71	97.74	3.01	483.10 ± 10.41	96.62	2.15
HQC	900.00	916.71 ± 23.24	101.86	2.54	909.02 ± 6.92	101.00	0.76

(b) Urine							
Spiked concentration in urine (ng/mL)		Andrographolide (AP1)			14-Deoxy-11,12-didehydroandrographolide (AP3)		
		Measured concentration (ng/mL)	(%) Accuracy	(%) CV	Measured concentration (ng/mL)	(%) Accuracy	(%) CV
*Intra-day*							
LLOQ	0.98	0.93 ± 0.12	95.07	13.16	1.03 ± 0.14	104.95	13.24
LQC	2.50	2.44 ± 0.19	97.74	7.70	2.36 ± 0.22	94.36	9.37
MQC	500.00	482.85 ± 19.17	96.57	3.97	471.55 ± 20.17	94.31	4.28
HQC	900.00	907.89 ± 34.15	100.88	3.76	899.33 ± 49.11	99.93	5.46
*Inter-day*							
LLOQ	0.98	1.00 ± 0.06	102.39	6.19	0.94 ± 0.07	96.61	7.62
LQC	2.50	2.52 ± 0.07	100.86	2.86	2.47 ± 0.10	98.91	4.05
MQC	500.00	486.29 ± 3.52	97.26	0.72	492.26 ± 19.97	98.45	4.06
HQC	900.00	903.87 ± 3.69	100.43	0.41	899.67 ± 16.36	99.97	1.82

Spiked concentration in urine (ng/mL)		Neoandrographolide (AP4)			14-Deoxyandrographolide (AP6)		
		Measured concentration (ng/mL)	(%) Accuracy	(%) CV	Measured concentration (ng/mL)	(%) Accuracy	(%) CV
*Intra-day*							
LLOQ	0.98	0.97 ± 0.15	99.16	15.07	0.93 ± 0.14	94.92	14.82
LQC	2.50	2.42 ± 0.72	96.70	7.26	2.46 ± 0.19	98.29	7.56
MQC	500.00	459.79 ± 16.29	91.96	3.54	483.46 ± 28.74	96.69	5.94
HQC	900.00	920.82 ± 33.39	102.31	3.63	912.96 ± 35.26	101.44	3.86
*Inter-day*							
LLOQ	0.98	0.98 ± 0.05	99.77	5.33	0.97 ± 0.06	99.24	6.22
LQC	2.50	2.46 ± 0.09	98.21	3.54	2.50 ± 0.05	99.89	2.10
MQC	500.00	483.41 ± 20.49	96.68	4.24	491.63 ± 8.36	98.33	1.70
HQC	900.00	904.97 ± 15.66	100.55	1.73	912.86 ± 4.42	101.43	0.48

Data expressed as mean ± SD, (n = 5).

Lower limit of quantitation (LLOQ).

Low quality control concentration (LQC concentration), medium quality control concentration (MQC concentration), high quality control concentration (HQC concentration).

#### Linearity, range and lower limit of quantification (LLOQ)

Three calibration curves were calculated based on data from three consecutive days using twelve concentrations for each standard in plasma and urine. The coefficients of variation of AP1, AP3, AP4, and AP6, were 0.24–9.76, 0.58–11.48, 0.20–10.64, 0.53–10.26 for plasma, and 2.71–13.43, 2.57–10.46, 2.55–10.13, 1.69–10.88 for urine, respectively. Percent accuracies were 85.50–109.02%, 94.26–106.46%, 90.93–101.04%, 92.75–110.08% for plasma, and 91.14–103.01%, 92.41–106.39%, 90.39–105.42%, 91.22–110.17% for urine, respectively. The results show good linearity (> 0.99) for both plasma and urine over the range of 0.98–1000.00 ng/mL. This method established LLOQ for the four diterpenoids at 0.98 ng/mL in both plasma and urine. (Supporting information, Tables S3 and S4).

#### Recovery

Mean extraction recovery for each analyte and IS in plasma and urine is summarized in Table 4a, b. The results indicate that analyses of urine samples exhibited higher recovery compared to plasma samples (78.42–89.72% for plasma and 81.63–95.09% for urine), %CV for plasma and urine was within the range of 2.13–13.23%.

**Table 4:** Mean extraction recovery, matrix effect, and hemolyzed plasma effect for andrographolide (AP1); 14-deoxy-11, 12-didehydroandrographolide (AP3); neoandrographolide (AP4); and 14-deoxyandrographolide (AP6) in plasma (a) and in urine (b) samples.

Compounds	Concentration (ng/mL)		Recovery		Matrix effect			Hemolyzed plasma effect	
			% Recovery	% CV	% CV	Matrix factor	IS normalized MF	% Accuracy	% CV
*(a) Plasma*									
Andrographolide	LQC	2.50	78.42	11.37	2.37	0.82	0.91	98.64	3.69
	MQC	500.00	81.64	2.13	7.70	0.93	1.01	99.14	5.26
	HQC	900.00	81.07	7.34	8.96	0.89	0.97	100.07	2.33
14-Deoxy-11,12-didehydroandrographolide	LQC	2.50	81.65	10.25	6.32	0.84	0.92	99.32	4.03
	MQC	500.00	82.95	8.72	5.58	0.90	0.98	102.83	5.12
	HQC	900.00	89.72	4.28	2.08	0.88	0.96	99.85	1.54
Neoandrographolide	LQC	2.50	79.21	13.04	4.01	0.75	0.83	98.76	7.10
	MQC	500.00	85.36	4.34	4.23	0.80	0.87	101.48	2.90
	HQC	900.00	82.45	9.65	1.87	0.83	0.91	100.93	1.12
14-Deoxyandrographolide	LQC	2.50	87.50	9.89	5.99	0.87	0.96	97.46	5.73
	MQC	500.00	85.44	5.42	4.14	0.90	0.98	99.98	2.86
	HQC	900.00	84.86	9.76	3.00	0.87	0.96	99.31	1.72
*(b) Urine*									
Andrographolide	LQC	2.50	81.63	8.88	5.12	0.84	0.95		
	MQC	500.00	88.43	6.70	6.34	0.91	1.01		
	HQC	900.00	91.80	8.20	7.03	0.89	1.10		
14-Deoxy-11,12-didehydroandrographolide	LQC	2.50	82.87	13.23	7.58	0.84	0.95		
	MQC	500.00	88.12	5.54	4.17	1.04	1.15		
	HQC	900.00	84.82	10.55	0.50	0.96	1.19		
Neoandrographolide	LQC	2.50	86.06	9.49	10.78	0.82	0.93		
	MQC	500.00	84.40	10.27	3.67	0.84	0.93		
	HQC	900.00	84.16	6.88	3.97	0.83	1.03		
14-Deoxyandrographolide	LQC	2.50	95.09	7.08	7.74	0.87	0.99		
	MQC	500.00	85.09	7.30	7.06	0.82	0.91		
	HQC	900.00	85.21	8.58	13.48	0.90	1.11		

Data expressed as mean ± SD, (n = 5).

Low quality control concentration (LQC concentration), medium quality control concentration (MQC concentration), high quality control concentration (HQC concentration).

#### Matrix effects

The matrix effects at three levels of QC concentrations are presented in Table 4a, b. The matrix effect indicated as the IS normalized MF of each analyte ranged between 0.83–1.01 for plasma and 0.91–1.19 for urine, indicating that the matrix effect of this newly developed method was negligible (IS normalized MF values were within 0.8–1.2). As shown in Table 4, %CVs were within the acceptance criteria (≤ 15%).

#### Stability

The stability of the four major diterpenoids in biological matrices was evaluated under different conditions. The results showed that AP1, AP3, AP4 and AP6 were stable in plasma and urine during five cycles of freeze–thaw storage. The analytes were also found to be stable after 3 months of “long-term” storage at − 80 °C. The extracted samples kept in the autosampler (at 4 °C) were stable for at least 24 h. No significant levels of degradation products were detected during the sample preparation process. The samples were stable for up to 8 h at room temperature as determined by quantitative analysis of all four analytes. The stability results in plasma and urine are summarized in Table 5a, b, respectively.

**Table 5:** Stability of andrographolide (AP1); 14-deoxy-11, 12-didehydroandrographolide (AP3); neoandrographolide (AP4); and 14-deoxyandrographolide (AP6) in (a) human plasma and (b) urine under different storage conditions.

(a) Plasma				
Storage conditions	Concentration (ng/mL)	Measured concentration (ng/mL, mean ± SD)	%Accuracy	%CV
*Andrographolide (AP1)*				
Short term stability 8 h at room temperature	2.50 (LQC)	2.34 ± 0.15	93.65	6.36
	500.00 (MQC)	445.29 ± 9.56	89.06	2.15
	900.00 (HQC)	888.87 ± 23.16	98.76	2.61
Freeze–thaw stability 5 cycles	2.50 (LQC)	2.45 ± 0.06	97.99	2.30
	500.00 (MQC)	465.07 ± 25.39	93.01	5.46
	900.00 (HQC)	857.48 ± 19.46	95.28	2.27
Long term stability 3 months at − 80 °C	2.50 (LQC)	2.36 ± 0.06	94.28	2.53
	500.00 (MQC)	458.87 ± 8.67	91.77	1.89
	900.00 (HQC)	893.21 ± 22.77	99.25	2.55
Post preparative stability 24 h at autosampler	2.50 (LQC)	2.35 ± 0.18	93.81	7.45
	500.00 (MQC)	463.01 ± 10.97	92.60	2.37
	900.00 (HQC)	895.66 ± 31.23	99.52	3.49
*14-Deoxy-11,12-didehydroandrographolide (AP3)*				
Short term stability 8 h at room temperature	2.50 (LQC)	2.44 ± 0.04	97.61	1.65
	500.00 (MQC)	472.00 ± 19.37	94.40	4.10
	900.00 (HQC)	887.83 ± 21.04	98.65	2.37
Freeze–thaw stability 5 cycles	2.50 (LQC)	2.56 ± 0.21	102.35	8.31
	500.00 (MQC)	466.24 ± 38.67	93.25	8.30
	900.00 (HQC)	888.74 ± 16.64	98.75	1.87
Long term stability 3 months at − 80 °C	2.50 (LQC)	2.18 ± 0.05	87.13	2.19
	500.00 (MQC)	471.59 ± 4.35	94.32	0.92
	900.00 (HQC)	883.01 ± 16.08	98.11	1.82
Post preparative stability 24 h at autosampler	2.50 (LQC)	2.44 ± 0.04	97.41	1.65
	500.00 (MQC)	471.70 ± 15.57	94.34	3.30
	900.00 (HQC)	901.09 ± 11.50	100.12	1.28
*Neoandrographolide (AP4)*				
Short term stability 8 h at room temperature	2.50 (LQC)	2.45 ± 0.19	98.11	7.54
	500.00 (MQC)	474.29 ± 22.98	94.85	4.85
	900.00 (HQC)	890.60 ± 51.25	98.96	5.75
Freeze–thaw stability 5 cycles	2.50 (LQC)	2.49 ± 0.14	99.77	5.59
	500.00 (MQC)	470.07 ± 13.89	94.01	2.95
	900.00 (HQC)	906.60 ± 9.37	100.73	1.03
Long term stability 3 months at − 80 °C	2.50 (LQC)	2.33 ± 0.12	93.19	5.27
	500.00 (MQC)	473.25 ± 26.27	94.65	5.55
	900.00 (HQC)	883.06 ± 16.19	98.12	1.83
Post preparative stability 24 h at autosampler	2.50 (LQC)	2.57 ± 0.12	102.61	4.62
	500.00 (MQC)	485.59 ± 17.11	97.12	3.52
	900.00 (HQC)	885.84 ± 17.63	98.43	1.99
*14-Deoxyandrographolide (AP6)*				
Short term stability 8 h at room temperature	2.50 (LQC)	2.54 ± 0.21	101.65	8.10
	500.00 (MQC)	452.37 ± 10.16	90.47	2.25
	900.00 (HQC)	906.41 ± 47.83	100.71	5.28
Freeze–thaw stability 5 cycles	2.50 (LQC)	2.42 ± 0.04	96.87	1.53
	500.00 (MQC)	473.93 ± 25.58	94.79	5.40
	900.00 (HQC)	890.41 ± 25.49	98.93	2.86
Long term stability 3 months at − 80 °C	2.50 (LQC)	2.22 ± 0.09	88.89	3.95
	500.00 (MQC)	462.16 ± 14.34	92.43	3.10
	900.00 (HQC)	890.25 ± 20.07	98.92	2.26
Post preparative stability 24 h at autosampler	2.50 (LQC)	2.52 ± 0.08	100.85	3.27
	500.00 (MQC)	481.96 ± 2.97	96.39	0.62
	900.00 (HQC)	911.38 ± 18.09	101.27	1.99

(b) Urine				
Storage conditions	Concentration (ng/mL)	Measured concentration (ng/mL, mean ± SD)	%Accuracy	%CV
*Andrographolide (AP1)*				
Short term stability 8 h at room temperature	2.50 (LQC)	2.33 ± 0.06	93.15	2.40
	500.00 (MQC)	479.00 ± 32.64	95.80	6.81
	900.00 (HQC)	916.95 ± 32.00	101.88	3.49
Freeze–thaw stability 5 cycles	2.50 (LQC)	2.36 ± 0.20	94.39	8.47
	500.00 (MQC)	470.05 ± 32.15	94.01	6.84
	900.00 (HQC)	889.00 ± 36.63	98.78	4.12
Long term stability 3 months at -80 °C	2.50 (LQC)	2.38 ± 0.16	95.31	3.87
	500.00 (MQC)	458.89 ± 20.53	91.78	4.47
	900.00 (HQC)	882.74 ± 14.22	98.08	1.61
Post preparative stability 24 h at autosampler	2.50 (LQC)	2.36 ± 0.18	94.23	7.56
	500.00 (MQC)	474.97 ± 23.30	95.00	4.91
	900.00 (HQC)	896.96 ± 17.62	99.66	1.97
*14-Deoxy-11,12-didehydroandrographolide (AP3)*				
Short term stability 8 h at room temperature	2.50 (LQC)	2.36 ± 0.07	94.35	3.11
	500.00 (MQC)	453.16 ± 19.26	90.63	4.25
	900.00 (HQC)	916.89 ± 26.49	101.88	2.89
Freeze–thaw stability 5 cycles	2.50 (LQC)	2.35 ± 0.10	93.92	4.06
	500.00 (MQC)	459.54 ± 15.54	91.91	3.38
	900.00 (HQC)	901.72 ± 35.81	100.19	3.97
Long term stability 3 months at − 80 °C	2.50 (LQC)	2.36 ± 0.06	94.28	2.53
	500.00 (MQC)	451.82 ± 15.00	90.36	3.32
	900.00 (HQC)	883.61 ± 32.83	98.18	3.72
Post preparative stability 24 h at autosampler	2.50 (LQC)	2.34 ± 0.11	93.61	4.85
	500.00 (MQC)	451.66 ± 9.81	90.33	2.17
	900.00 (HQC)	902.47 ± 52.60	100.28	5.83
*Neoandrographolide (AP4)*				
Short term stability 8 h at room temperature	2.50 (LQC)	2.43 ± 0.07	97.15	3.06
	500.00 (MQC)	461.15 ± 18.24	92,229.00	3.96
	900.00 (HQC)	925.37 ± 32.74	102.82	3.54
Freeze–thaw stability 5 cycles	2.50 (LQC)	2.35 ± 0.08	93.80	3.48
	500.00 (MQC)	464.68 ± 10.80	92.94	2.32
	900.00 (HQC)	910.07 ± 23.86	101.12	2.62
Long term stability 3 months at − 80 °C	2.50 (LQC)	2.34 ± 0.16	93.48	6.95
	500.00 (MQC)	445.65 ± 10.92	89.13	2.45
	900.00 (HQC)	852.94 ± 7.53	94.77	0.88
Post preparative stability 24 h at autosampler	2.50 (LQC)	2.40 ± 0.15	96.03	6.18
	500.00 (MQC)	449.68 ± 2.36	89.94	0.52
	900.00 (HQC)	916.25 ± 29.10	101.81	3.18
*14-Deoxyandrographolide (AP6)*				
Short term stability 8 h at room temperature	2.50 (LQC)	2.39 ± 0.15	95.56	6.09
	500.00 (MQC)	459.52 ± 16.67	91.90	3.63
	900.00 (HQC)	921.74 ± 21.18	102.42	2.30
Freeze–thaw stability 5 cycles	2.50 (LQC)	2.39 ± 0.18	95.48	7.67
	500.00 (MQC)	470.16 ± 19.65	94.03	4.18
	900.00 (HQC)	924.91 ± 7.49	102.77	0.81
Long term stability 3 months at − 80 °C	2.50 (LQC)	2.35 ± 0.07	93.89	3.12
	500.00 (MQC)	454.38 ± 21.26	90.88	4.68
	900.00 (HQC)	893.39 ± 24.76	99.27	2.77
Post preparative stability 24 h at autosampler	2.50 (LQC)	2.41 ± 0.13	96.20	5.34
	500.00 (MQC)	469.68 ± 35.58	93.94	7.58
	900.00 (HQC)	911.80 ± 10.76	101.31	1.18

Data expressed as mean ± SD (n = 5).

Low quality control concentration (LQC concentration), medium quality control concentration (MQC concentration), high quality control concentration (HQC concentration).

#### Hemolyzed plasma effects

There was no interference observed in the hemolyzed plasma blank at the retention time of each analyte and of IS. As indicated in Table 4, the %CVs of AP1, AP3, AP4 and AP6 were less than 15% (1.12–7.10%) and the %accuracy of LQC and HQC samples was within a range of 97.46–100.93%.

### Quantitative determination of conjugated compounds in plasma and urine using enzymatic digestion assay

**Figure 2:**
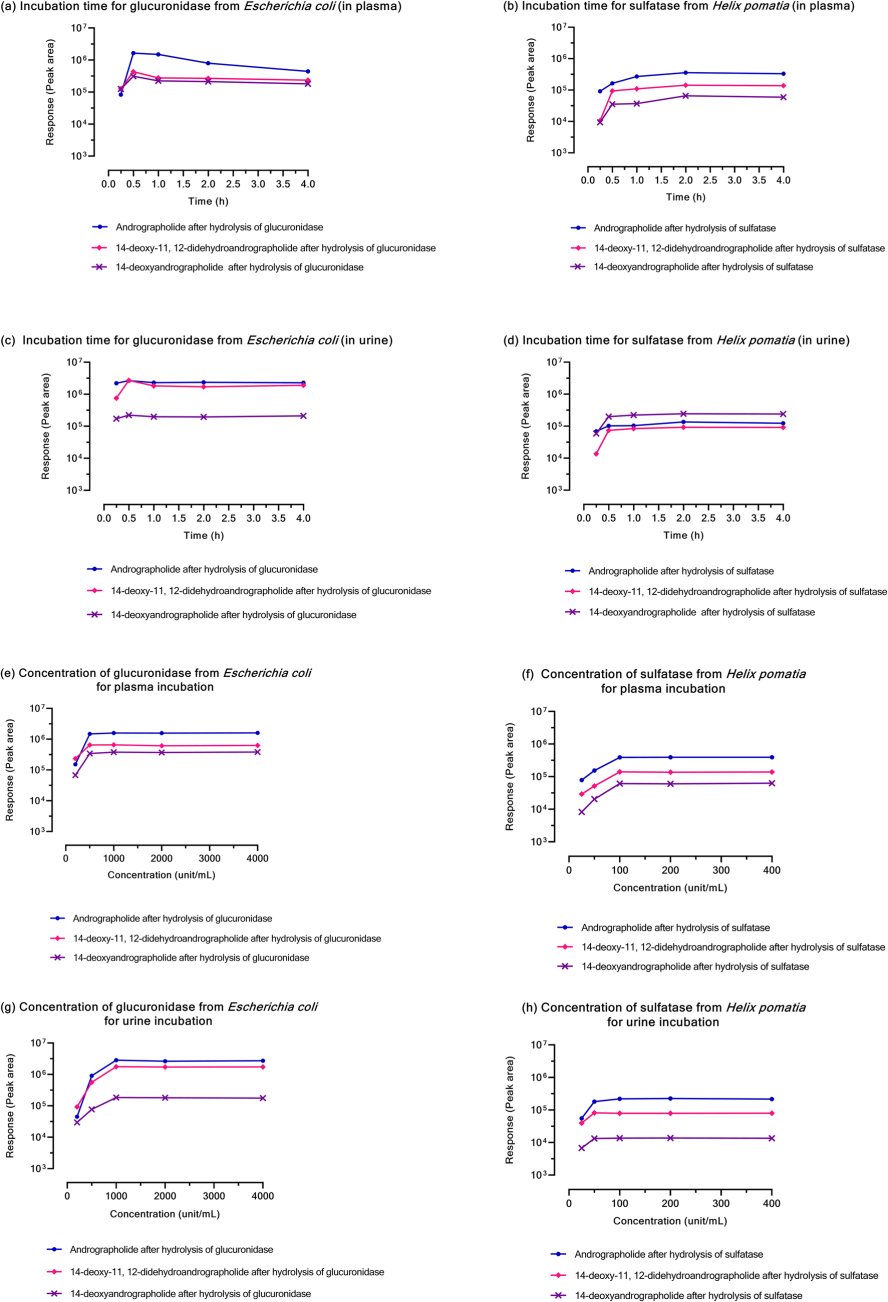
**(a–h)** Peak area ratio of four major diterpenoids with incubation time **(a–d)** and enzyme concentrations **(e–h)** after hydrolysis of *β*-glucuronidase type IX-A from *Escherichia coli*, glucuronidase activity of 1,000,000–5,000,000 units/g protein (glucuronidase activity = 2,354,185 units/g protein); and after hydrolysis of sulfatase*,* type H-1 from *Helix pomatia*, sulfatase activity ≥ 10,000 units/g solid (sulfatase activity = 16,134 units/g solid and *β*-glucuronidase activity = 353,820 units/g solid).

The method for deconjugation was developed and optimized to obtain a complete hydrolysis reaction in plasma and urine samples. The results indicate that incubation time and enzyme concentration influence the hydrolysis reaction as observed in the measured concentration of each compound (Fig. 2a–h).

The highest completed hydrolysis of *β*-glucuronidase in phosphate buffer medium was obtained in 30 min (Fig. 2a, c), whereas sulfatase in acetate buffer completed the hydrolysis reaction within 2 h (Fig. 2b, d). The hydrolysis of each conjugated metabolite was increased when the samples were incubated with 250–1000 units/mL of glucuronidase and 25–100 units/mL of sulfatase (Fig. 2e–h). The use of higher concentrations of enzyme did not further increase hydrolysis, the reaction remained at a plateau at 1000–4000 units/mL of glucuronidase and 100–400 units/mL of sulfatase. Therefore, this study used 1000 units/mL of *β*-glucuronidase (type IX-A) from *Escherichia coli* and 100 units/mL of sulfatase type H-1 from *Helix pomatia* (containing sulfatase 100 units/mL and glucuronidase 2193 units/mL).

Finally, the hydrolysis of glucuronide metabolites in the pharmacokinetic study was performed according to the following process: 50 µL of plasma or urine sample was incubated for 30 min with 50 µL of 1000 U/mL *β*-glucuronidase in phosphate buffer pH 6.8 (composed of 0.1 M sodium phosphate monobasic and 0.1 M sodium phosphate dibasic) at 37 °C. For the hydrolysis of sulfatase, 50 µL of 100 units/mL of sulfatase in an acetate buffer at pH 5.0 (composed of 0.1 M acetic acid and 0.1 M sodium acetate) was added into 50 µL of sample; the mixture was then incubated at 37 °C for 2 h. The reaction was stopped in all hydrolysis samples with 150 µL of cold methanol, and then protein precipitation was performed prior to LC–MS/MS analysis. However, due to unavailable standard of the conjugated forms (AP-glucuronide and sulfate-glucuronide). The quantitative analysis of conjugated metabolites was determined after the hydrolysis reaction by enzymatic digestion, and the free form was analyzed without hydrolysis. The conjugated standards of AP-glucuronide or AP-sulfate should be synthesized for determining the exact amount of the glucuronide or sulfate.

In this study, the concentrations of the total conjugated metabolites were used to generate plasma and urine concentration–time profiles. Standard curves were calculated for both plasma and urine samples and were within a range of 0.98–1000.00 ng/mL. The coefficients of variation of AP1, AP3, AP4, and AP6, were within 1.60–7.18%, 1.04–9.84%, 3.84–10.49%, 2.80–12.03%, for plasma; and 2.76–8.84%, 4.05–11.76%, 2.65–9.67%, 3.07–10.78%, for urine, respectively. Percentage of accuracies were within 92.98–107.22%, 92.25–107.47%, 94.11–103.41%, 97.73–107.46% for plasma, and 92.74–105.31%, 92.10–105.98%, 91.24–103.81%, 94.72–107.69% for urine, respectively.

### Application of the analytical method in a pharmacokinetic study

**Figure 3:**
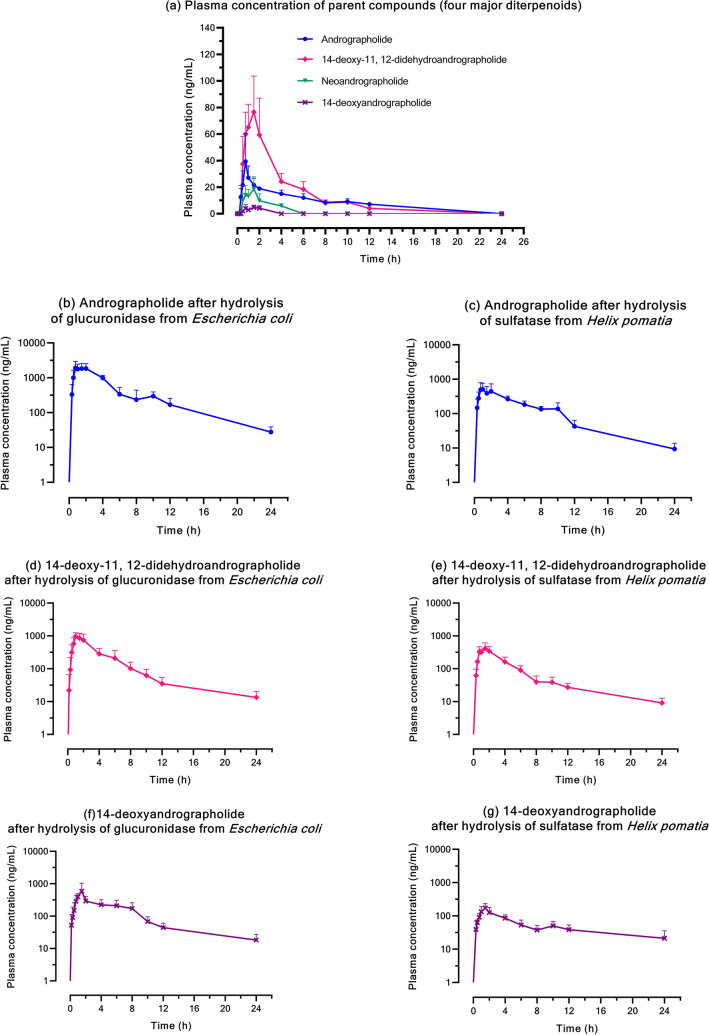
**(a–g)** Mean plasma concentration–time profiles following a single oral administration of *A. paniculata* extract; **(a)** Concentration of the four parent diterpenoids, and **(b–g)** concentration of each diterpenoid after hydrolysis of *β*-glucuronidase type IX-A from *Escherichia coli*, and sulfatase type H-1 from *Helix pomatia.*

Plasma concentration–time profiles of the four major diterpenoids (AP1, AP3, AP4, and AP6) and their respective conjugated metabolites after oral administration of a single dose of *A. paniculata* extract (calculated as 60 mg/dose of andrographolide) are shown in Fig. 3. The related pharmacokinetic parameters calculated by non-compartmental analysis are summarized in Table 6. Following a single-dose pharmacokinetic study, the four major diterpenoids; andrographolide (AP1), 14-deoxy-11, 12-didehydroandrographolide (AP3), neoandrographolide (AP4), and 14-deoxyandrographolide (AP6) were partially absorbed and then entered into the circulatory system. These four parent compounds (Fig. 3a) reached their peak plasma concentration within 0.80–1.50 h post dose (T_max_), while T_max_ parameters for the conjugated metabolites were observed within 1.25–1.50 h after dosing (Fig. 3b–g). Notably, a greater AUC was observed for both conjugated metabolites of AP1, AP3, and AP6, when compared to the respective parent compounds. By contrast, neoandrographolide (AP4) was detected in only very small amounts in plasma and its conjugated metabolites were negligible.

**Table 6:** Clinical pharmacokinetic parameters following oral administration of *A. paniculata* extract. (a) Parent compounds (AP1, AP3, AP4 and AP6); (b) apparent conjugated metabolite after hydrolysis of *β*-glucuronidase from *Escherichia coli* and (c) apparent conjugated metabolites after hydrolysis of sulfatase, type H-1 from *Helix pomatia.*

PK parameters	(a) Parent compounds (n = 4)		(b) Apparent conjugated glucuronide after hydrolysis of *β*-glucuronidase (n = 4)		(c) Apparent conjugated metabolites after hydrolysis of sulfatase* (n = 4)	
*Andrographolide (AP1)*						
C_max_ (µg/L)^a^	60.85 ± 26.52		2375.78 ± 837.90		630.98 ± 261.29	
T_max_ (h)^b^	0.80	[0.05]	1.50	[1.00]	1.50	[1.55]
AUC_(0−t)_ (µg-hr/L)^a^	209.6 ± 21.06		9647.03 ± 1362.70		2745.85 ± 701.37	
Vd/F (L/Kg)^a^	4.58 ± 3.28		0.25 ± 0.06		1.18 ± 0.48	
Half-life (h)^b^	1.27	[0.02]	4.97	[0.35]	4.63	[3.50]
*14-Deoxy-11,12-didehydroandrographolide (AP3)*						
C_max_ (µg/L)^a^	83.78 ± 22.25		1146.13 ± 185.36		382.35 ± 140.39	
T_max_ (h)^b^	1.50	[0.30]	1.25	[0.63]	1.50	[0.30]
AUC_(0−t)_ (µg-h/L)^a^	308.98 ± 59.02		3286.60 ± 960.14		1483.35 ± 400.65	
Vd/F (L/kg)^a^	0.73 ± 0.10		1.03 ± 1.07		0.93 ± 0.13	
Half-life (h)^b^	1.38	[0.02]	11.58	[17.16]	8.86	[1.89]
*Neoandrographolide (AP4)*						
C_max_ (µg/L)^a^	22.35 ± 5.87		N/A		N/A	
T_max_ (h)^b^	1.50	[0.30]	N/A		N/A	
AUC_(0−t)_ (µg-hr/L)^a^	44.13 ± 7.48		N/A		N/A	
Vd/F (L/Kg)^a^	0.65 ± 0.13		N/A		N/A	
Half-life (h)^b^	0.22	[0.02]	N/A		N/A	
*14-Deoxyandrographolide (AP6)*						
C_max_ (µg/L)^a^	6.33 ± 1.18		657.55 ± 405.09		189.75 ± 56.57	
T_max_ (h)^b^	1.50	[0.30]	1.25	[0.63]	1.25	[0.50]
AUC_(0−t)_ (µg-hr/L)^a^	10.18 ± 2.90		2690.45 ± 775.82		1179.63 ± 356.50	
Vd/F (L/kg)^a^	1.93 ± 1.03		0.25 ± 0.13		0.58 ± 0.04	
Half-life (h)^b^	0.23	[0.01]	8.67	[4.44]	14.51	[8.54]

* apparent conjugated metabolites after hydrolysis of sulfatase, type H-1 from *Helix pomatia* (containing sulfatase and glucuronidase activities).

C_max_, maximum plasma concentration; T_max_, time to reach maximum concentration; AUC, area under the plasma concentration–time curve; Vd/F, the apparent volume of distribution; Half-life, elimination half-life.

N/A = not applicable.

^a^Data are expressed as mean ± SD (n = 4).

^b^Data is expressed as median [IQR].

**Figure 4:**
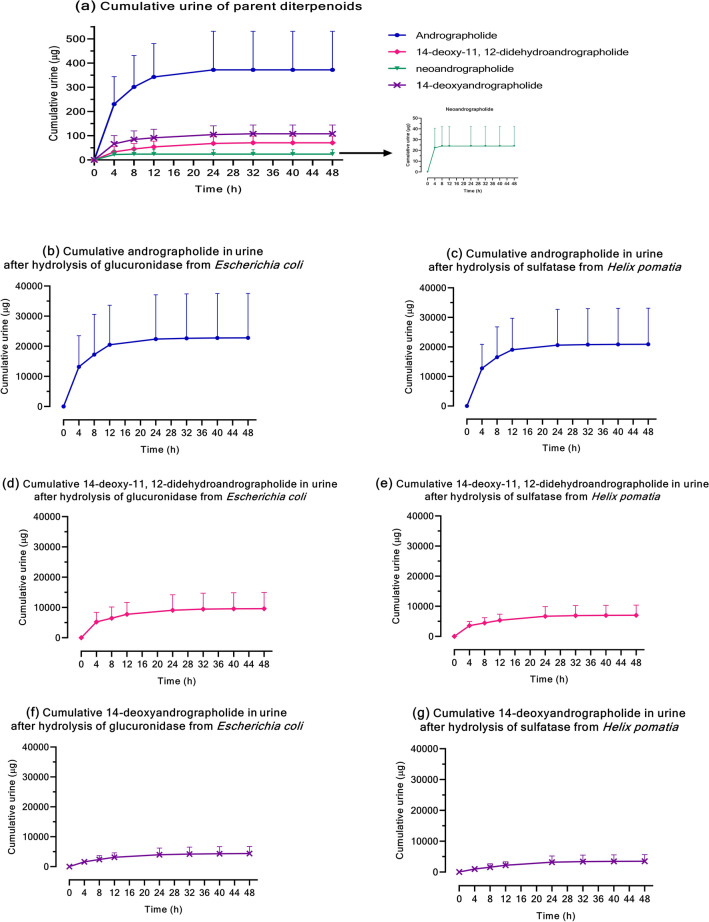
**(a–g)** Cumulative urine excretion following a single oral administration of *A. paniculata* extract. **(a)** Cumulative urine of the four parent diterpenoids, and **(b–g)** cumulative urine of each diterpenoid after hydrolysis of β-glucuronidase type IX-A from *Escherichia coli*, and sulfatase type H-1 from *Helix pomatia*.

Regarding the termination phase after oral administration of *A. paniculata* extract, all four major compounds (AP1, AP3, AP4 and AP6) of the extract were primarily excreted renally. A portion of these parent compounds underwent biotransformation in the liver to become the conjugated metabolites that were also eliminated via the urine. The exception to this was neoandrographolide (AP4). Similar to the results in plasma, conjugated metabolites of AP4 were negligible in the urine. The accumulation of parent diterpenoids in urine occurred within 32 h after administration (Fig. 4a), whereas the conjugated metabolites were intensively eliminated through the kidney over 48 h post-dose (Fig. 4b–g). Considering the pharmacokinetic parameters of excretion, the conjugated metabolites were retained in human blood circulation longer than their respective parent compounds, as evidenced by the longer half-life of the metabolites (Table 6).

## Discussion

In a review of the literature, most studies determined only andrographolide^11–13^, while other major active diterpenoids of *A. paniculata* extract were not investigated during pharmacokinetic studies. Although the previous method could analyze the major active diterpenoids^6^, it restricts the sample matrix to only plasma with a quantifiable concentration of LLOQ (2.5 ng/mL)^6^. Due to these limitations, a bioanalytical method for quantitative determination of all four major diterpenoids, in both plasma and urine, was developed and validated for application in pharmacokinetic study. The method was completely validated at the quantifiable limit of LLOQ = 0.98 ng/mL. The plasma concentration of andrographolide was detectable until a completion of the elimination phase. This method minimized the sample volume necessary for extraction. The additional process was expanded to cover not only quantification of parent compounds but also of their conjugated metabolites in plasma and urine by hydrolysis reaction. In addition, this method also shortens the run time required for chromatographic separation to 6 min on the High-Performance Liquid Chromatography (HPLC). It is, therefore, beneficial to use this analytical method for the quantification of major parent compounds and conjugated metabolites of *A. paniculata* extract in biological samples, even when using a limited volume of plasma or urine obtained in animal or human studies. Moreover, this newly developed method is useful in clinical pharmacokinetics, when a large number of samples must be analyzed in a short time period.

The validated analytical method in this study was applied to determine four active diterpenoids in preliminary testing of four subject samples. The results of the pilot pharmacokinetic study of 4 subjects show that the four major parent compounds are partially absorbed into blood circulation, resulting in low detectable concentrations of the free form of each analyte in plasma samples. The analysis of plasma levels of the parent compounds increased after deconjugation by glucuronidase and sulfatase enzymes, suggesting the presence of glucuronide and sulfate metabolites. This finding implies that after oral administration of *A. paniculata* extract, the parent compounds were extensively biotransformed by phase II hepatic metabolic pathways, where the compounds were conjugated with glucuronic acid and sulfate to increase their polarity. These conjugated metabolites then entered into blood circulation and finally were eliminated partly through renal excretion. These results are consistent with the findings of earlier preclinical pharmacokinetics studies in rats, in which sulfate metabolites were detected in both the livers and urine^14,19,20^. In addition, another pharmacokinetics study in humans demonstrated that glucuronide and sulfate conjugated metabolites were present in urine after oral administration of *A. paniculata* extract^21,22^.

**Figure 5:**
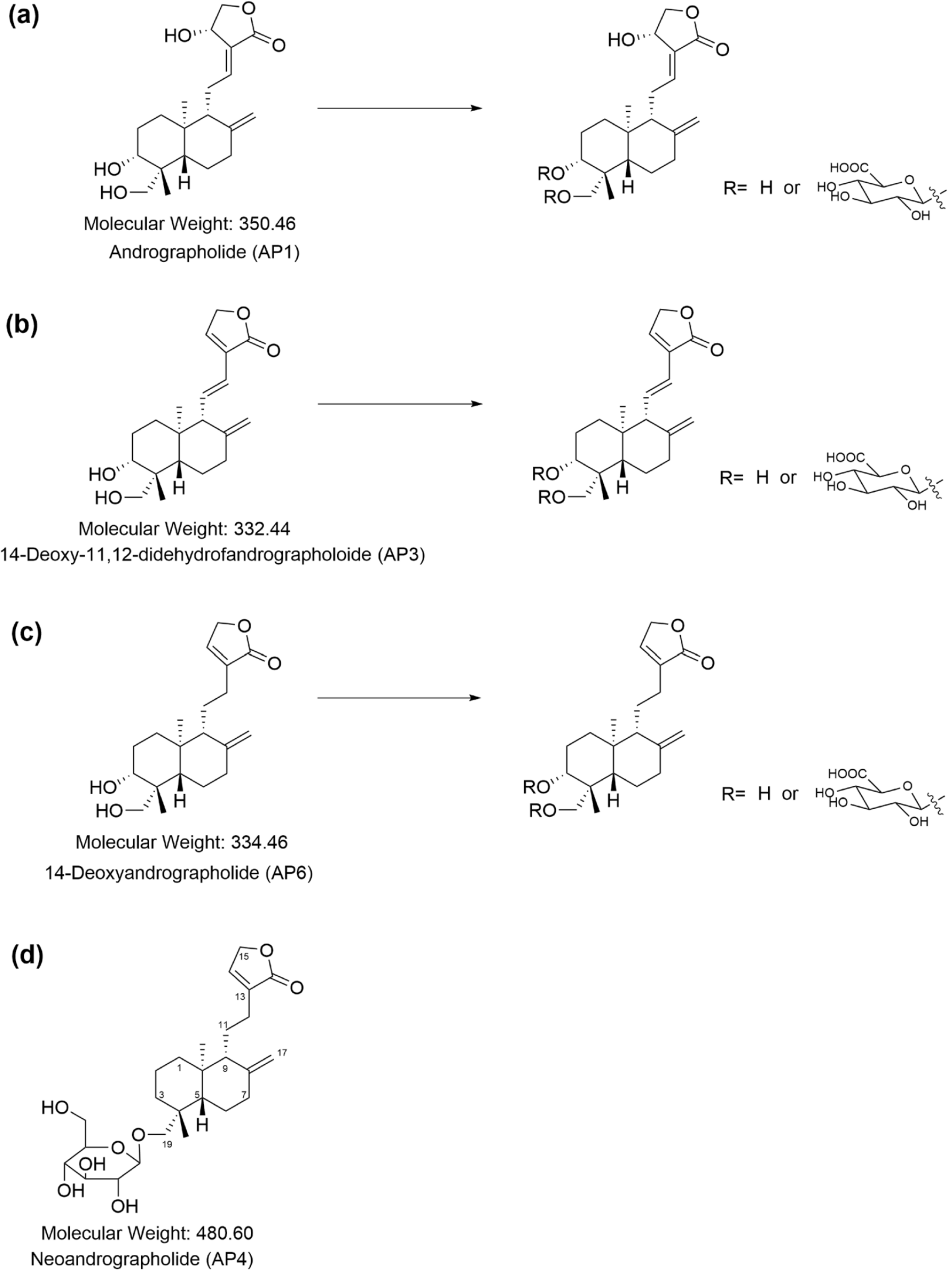
**(a–c)** The proposed metabolic pathways and chemical structures of major diterpenoids: andrographolide (AP1); 14-deoxy-11, 12-didehydroandrographolide (AP3);14-deoxyandrographolide (AP6); and their respective conjugated glucuronide metabolites. **(d)** Chemical structure of neoandrographolide (AP4).

**Figure 6:**
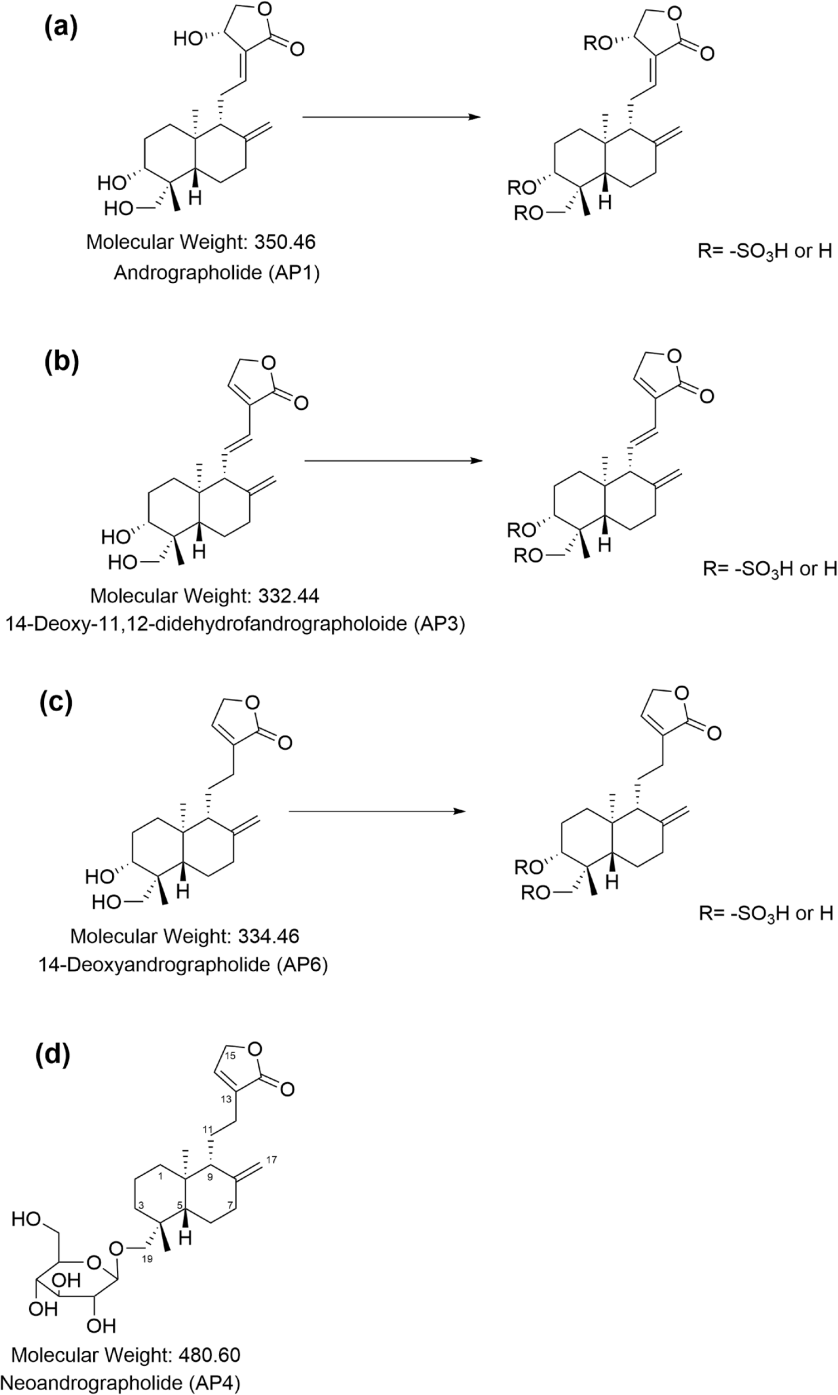
**(a–c)** The proposed metabolic pathways and chemical structures of major diterpenoids: andrographolide (AP1); 14-deoxy-11, 12-didehydroandrographolide (AP3); 14-deoxyandrographolide (AP6); and their respective conjugated sulfate metabolites. **(d)** Chemical structure of neoandrographolide (AP4).

Interestingly, plasma and urine concentrations of the conjugated metabolites of AP1, AP3, and AP6 were generally much higher than the concentrations of their corresponding parent compounds. The findings in this study strengthen those of a previous report indicating that the conjugated metabolite is likely one of the main metabolic pathways of *A. paniculata* extract. The one exception was the conjugation of neoandrographolide (AP4). The metabolites in terms of AP4 glucuronide and sulfate were negligible in plasma and urine samples. This finding suggests that glucuronidation and sulfation may not be the major metabolites of neoandrographolide. One reason for this may be that the chemical structure of neoandrographolide (diterpene glucoside) contains a sugar moiety at C-19 (Figs. 5d, 6d), which could have a steric hindrance effect that prevents the conjugation of glucuronic acid or sulfate at this position. The predicted metabolic pathways including the chemical structures of four parent diterpenoids and their tentative conjugated glucuronide or sulfate metabolites are proposed in Figs. 5a–c and 6a–c, respectively.

For elimination after administration of a single oral dose of *A. paniculata* extract, the highest cumulative amount of the unchanged parent compounds in urine was observed within 4 h after dosing. The changed forms in terms of conjugated glucuronide and sulfate metabolites of AP1, AP3 and AP6 were intensively excreted through the kidney from 4 h until 48 h post-dose.

However, the data presented in this clinical study were preliminary results. The pharmacokinetics of *A. paniculata* with a larger number of sample size have been currently investigated by using this analytical method. Another shortcoming of the present study involved the analysis of conjugated sulfate metabolite. The use of sulfatase type H-1 from *Helix pomatia*, which contained sulfatase and glucuronidase activities, resulted in the deconjugated forms of both sulfate and glucuronide. Therefore, it could not determine the exact amount of conjugated sulfate. Further studies may be required in order to carry out the entire hydrolysis of sulfatase.

## Conclusion

This newly developed LC–MS/MS method provides a thorough analytical method for the intended purposes of quantitative determination of the four major parent compounds (andrographolide; 14-deoxy-11, 12-didehydroandrographolide; neoandrographolide; and 14-deoxyandrographolide) in both plasma and urine samples. In this way, this method offers a solution to the limitations inherent in other methods and enables a more complete pharmacokinetic analysis. This highly sensitive method (LLOQ < 1.00 ng/mL), with requiring a short analysis time (6.0 min), was successfully applied in a pharmacokinetic study of orally administered *A. paniculata* extract in healthy subjects. Sample preparation was performed by protein precipitation and extraction by methanol, using a small sample volume (50 µL). Quantitative analysis of conjugated metabolites was determined using an enzyme hydrolysis reaction. The findings concerning the identification of novel metabolites of *A. paniculata* and the related compounds fill a gap in the knowledge of human metabolic pathways involved in their phase II biotransformation including glucuronidation and sulfation. Further, the findings of this pilot clinical pharmacokinetic study support future research efforts examining the use of *A. paniculata* extract in human (equivalent to andrographolide 60 mg) for the treatment of COVID-19 symptoms and other therapeutic uses.

## Supplementary Information

Supplementary Information.

## Abbreviations

AP1: Andrographolide
AP3: 14-Deoxy-11, 12-didehydroandrographolide
AP4: Neoandrographolide
AP6: 14-Deoxyandrographolide
AUC: Area under plasma concentration–time curve
BMI: Body mass index
C_max_: Maximum plasma concentration
CV: Coefficient of variation
ESI: Electrospray ionization
F: Absolute oral bioavailability
HPLC: High performance liquid chromatography
HQC concentration: High quality control concentration
IQR: Interquartile range
IS: Internal standard
LC–MS/MS: Liquid chromatography-tandem mass spectrometry
LC-QqQ-MS/MS: Liquid chromatography triple quadrupole tandem mass spectrometry
LC-QTOF/MS: Liquid chromatography-quadrupole time-of-flight mass spectrometry
LLOQ: Lower limit of quantification
LQC concentration: Low quality control concentration
MF: Matrix factor
MQC concentration: Medium quality control concentration
MRM: Multiple reaction monitoring
SD: Standard deviation
T_max_: Time to reach maximum plasma concentration
Half-life or t_1__/__2_: Elimination half-life
V_d_/F: Apparent volume of distribution after non-intravenous administration
