# Supplementary material for: A validated LC–MS/MS method for clinical pharmacokinetics and presumptive phase II metabolic pathways following oral administration of Andrographis paniculata extract
Source: Sci Rep. 2023 Feb 13;13:2534. doi: 10.1038/s41598-023-28612-1 (PMC9924185; doi:10.1038/s41598-023-28612-1)
Supplement: Supplementary file 1 — Supplementary Information. [file 41598_2023_28612_MOESM1_ESM.pdf]

A validated LC-MS/MS method for clinical pharmacokinetics and presumptive phase II metabolic pathways following oral administration of *Andrographis paniculata* extract

Phanit Songvut[1], Nanthanit Pholphana[1], Tawit Suriyo[1,2], Nuchanart Rangkadilok[1,2], Duangchit Panomvana[3], Porranee Puranajoti[3], Jutamaad Satayavivad[1,2*]

Affiliation

[1] Laboratory of Pharmacology, Chulabhorn Research Institute, Bangkok, Thailand

[2] Center of Excellence on Environmental Health and Toxicology (EHT), OPS, MHESI, Thailand

[3] Translational Research Unit, Chulabhorn Research Institute, Bangkok, Thailand

***Corresponding Author**

Assoc. Prof. Jutamaad Satayavivad, Ph.D.

Laboratory of Pharmacology, Chulabhorn Research Institute

54 Kamphaeng Phet 6 Rd., Laksi, Bangkok 10210, Thailand

Tel.: +66 2 553-8555 ext. 8539

Fax: +66 2 553-8526

E-mail address: jutamaad@cri.or.th

Supporting Information

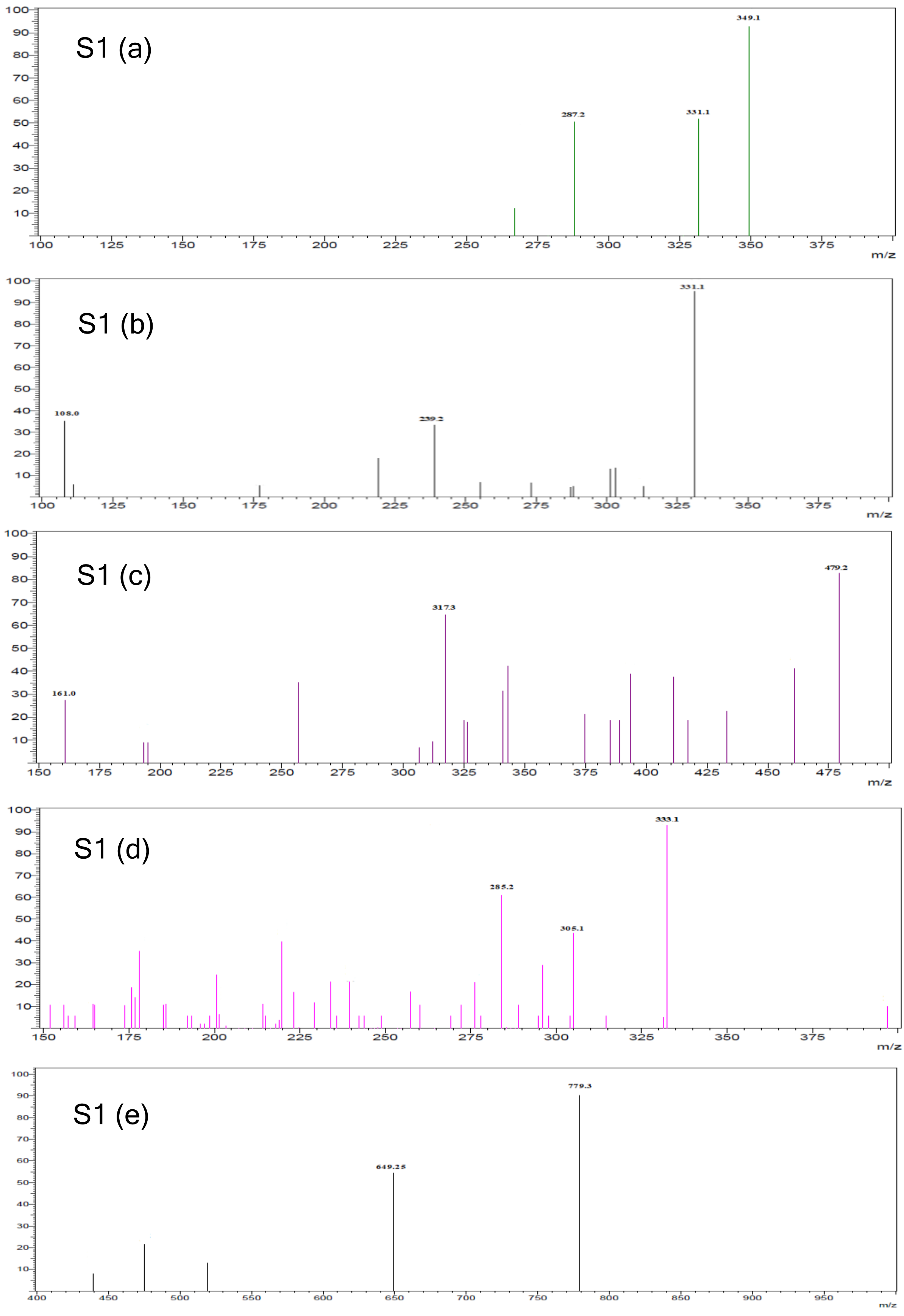

Figure S1: Mass spectrums of (**a**) andrographolide, AP1; **(b)** 14-deoxy-11,12-didehydroandrographolide, AP3; **(c)** neoandrographolide, AP4, **(d)** deoxyandrographolide, AP6; and **(e)** Digoxin, IS

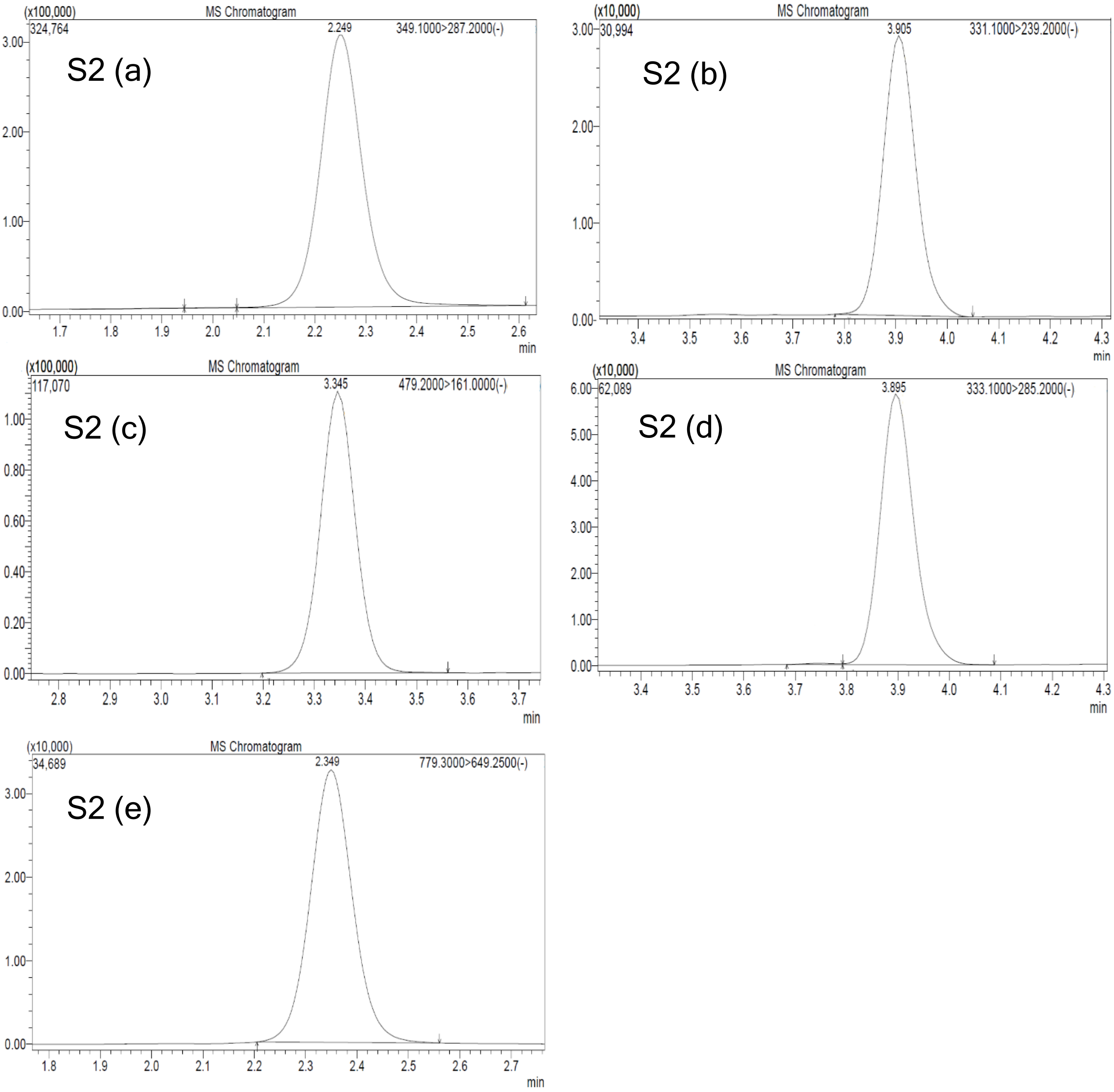

Figure S2: Chromatograms and retention times of **(a)** andrographolide, AP1; **(b)** 14-deoxy-11,12-didehydroandrographolide, AP3; **(c)** neoandrographolide, AP4, **(d)** deoxyandrographolide, AP6; and **(e)** Digoxin, IS; in methanol.

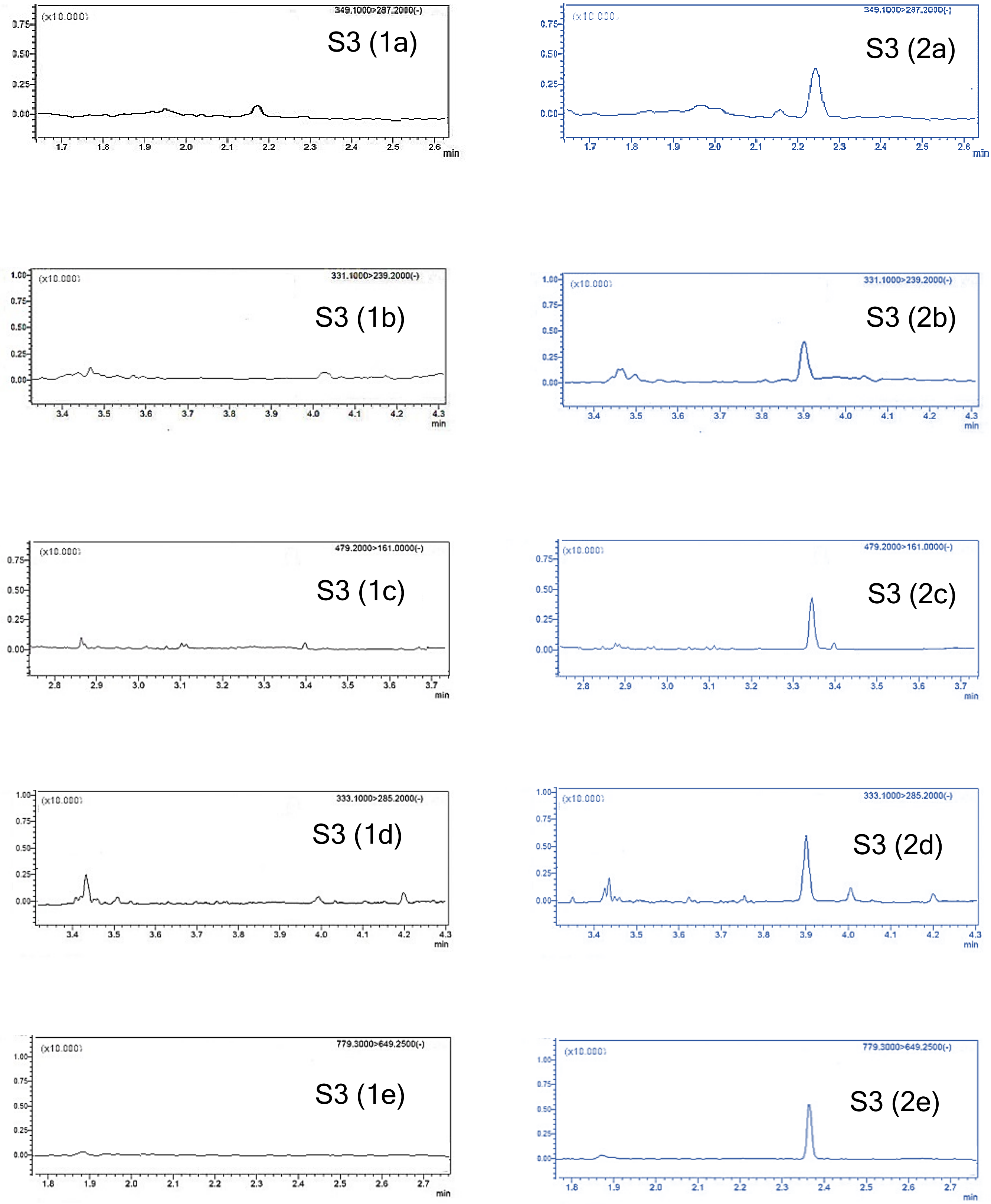

Figure S3: Chromatograms of **(1)** representative blank plasma and **(2)** spiked standards in blank plasma at LLOQ**; (a)** andrographolide, AP1; **(b)** 14-deoxy-11,12-didehydroandrographolide, AP3; **(c)** neoandrographolide, AP4, **(d)** deoxyandrographolide, AP6; and **(e)** Digoxin (IS).

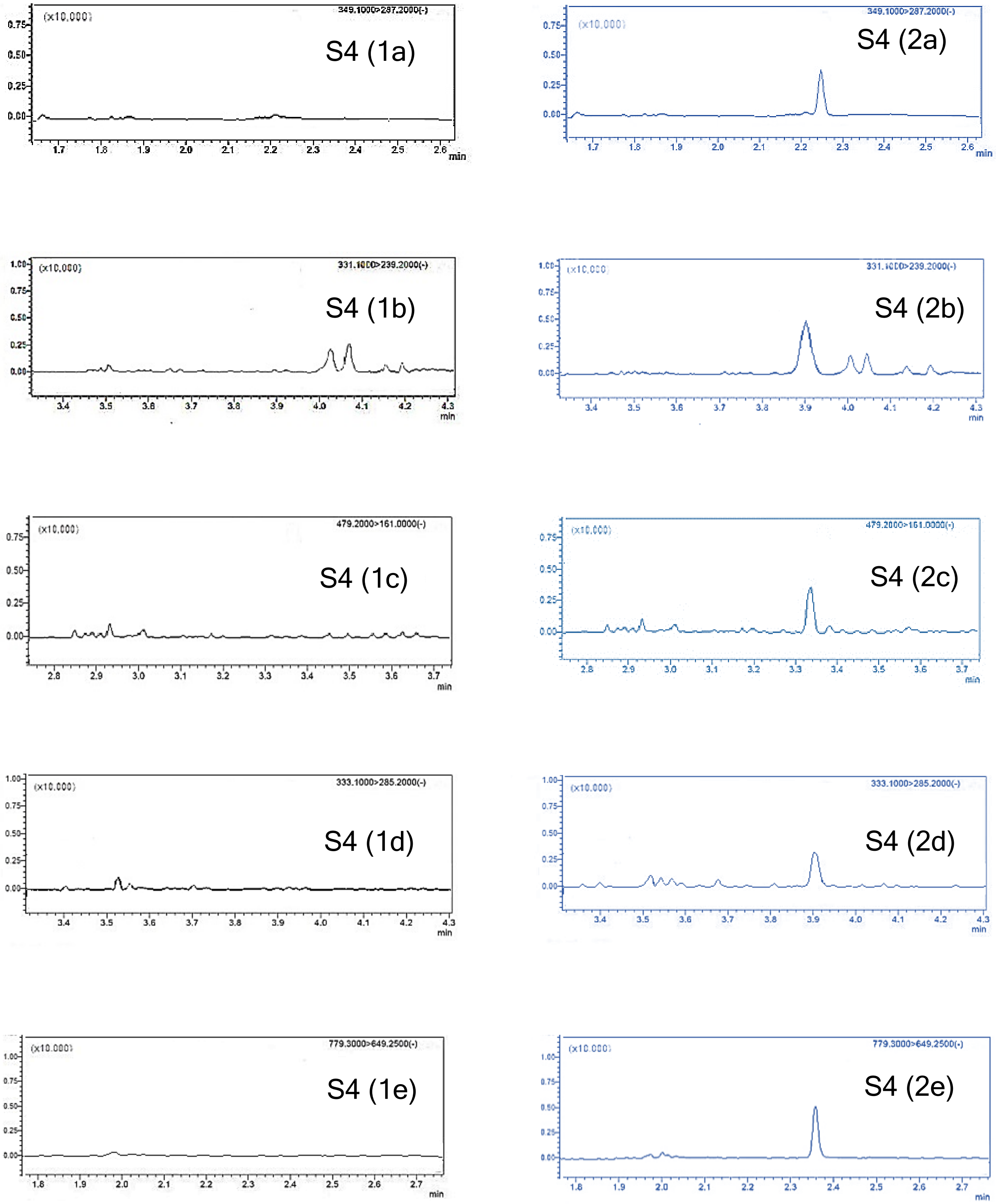

Figure S4: Chromatograms of **(1)** representative blank urine and **(2)** spiked standards in blank urine at LLOQ**;** **(a)** andrographolide, AP1; **(b)** 14-deoxy-11,12-didehydroandrographolide, AP3; **(c)** neoandrographolide, AP4, **(d)** deoxyandrographolide, AP6; and **(e)** Digoxin (IS).

Table S1: Accuracy and precision of andrographolide (AP1), 14-deoxy-11,12-didehydroandrographolide (AP3), neoandrographolide (AP4), and deoxyandrographolide, (AP6) in plasma

Andrographolide (AP1)				
Parameter	Concentration (ng/mL)			
	LLOQ	LQC	MQC	HQC
Mean of day 1	0.90	2.50	468.92	911.23
Mean of day 2	1.03	2.66	484.10	911.07
Mean of day 3	1.04	2.37	497.65	932.59
Average	0.99	2.51	483.56	918.30
SD	0.08	0.14	14.37	12.38
Precision (%CV)	7.97	5.66	2.97	1.35
% Accuracy	101.42	100.37	96.71	102.03
**14-deoxy-11,12-didehydroandrographolide (AP3)**				
Parameter	Concentration (ng/mL)			
	LLOQ	LQC	MQC	HQC
Mean of day 1	1.12	2.45	478.11	926.73
Mean of day 2	0.90	2.42	511.06	905.36
Mean of day 3	0.88	2.50	485.73	890.96
Average	0.97	2.46	491.63	907.68
SD	0.13	0.04	17.25	18.00
Precision (%CV)	13.43	1.50	3.51	1.98
% Accuracy	98.81	98.27	98.33	100.85
**Neoandrographolide (AP4)**				
Parameter	Concentration (ng/mL)			
	LLOQ	LQC	MQC	HQC
Mean of day 1	1.00	2.57	473.23	941.15
Mean of day 2	0.89	2.65	502.52	914.09
Mean of day 3	0.97	2.46	490.30	894.89
Average	0.95	2.56	488.68	916.71
SD	0.06	0.09	14.71	23.24
Precision (%CV)	5.99	3.68	3.01	2.54
% Accuracy	97.44	102.41	97.74	101.86
**Deoxyandrographolide (AP6)**				
Parameter	Concentration (ng/ml)			
	LLOQ	LQC	MQC	HQC
Mean of day 1	0.87	2.29	472.87	910.69
Mean of day 2	1.07	2.58	493.67	914.94
Mean of day 3	0.89	2.46	482.76	901.41
Average	0.94	2.44	483.10	909.02
SD	0.11	0.15	10.41	6.92
Precision (%CV)	11.35	6.06	2.15	0.76
% Accuracy	96.48	97.69	96.62	101.00

Table S2: Accuracy and precision of andrographolide (AP1), 14-deoxy-11,12-didehydroandrographolide (AP3), neoandrographolide (AP4), and deoxyandrographolide, (AP6) in urine

Andrographolide (AP1)				
**Parameter**	**Concentration (ng/mL)**			
	**LLOQ**	**LQC**	**MQC**	**HQC**
Mean of day 1	0.93	2.44	482.85	907.89
Mean of day 2	1.04	2.59	486.14	903.06
Mean of day 3	1.04	2.54	489.87	900.66
Average	1.00	2.52	486.29	903.87
SD	0.06	0.07	3.52	3.68
Precision (%CV)	6.19	2.86	0.72	0.41
% Accuracy	102.39	100.86	97.26	100.43
**14-deoxy-11,12-didehydroandrographolide (AP3)**				
**Parameter**	**Concentration (ng/mL)**			
	**LLOQ**	**LQC**	**MQC**	**HQC**
Mean of day 1	1.03	2.36	471.55	899.33
Mean of day 2	0.89	2.55	493.83	883.48
Mean of day 3	0.92	2.51	511.40	916.20
Average	0.94	2.47	492.26	899.67
SD	0.07	0.10	19.97	16.36
Precision (%CV)	7.62	4.05	4.06	1.82
% Accuracy	96.61	98.91	98.45	99.96
**Neoandrographolide (AP4)**				
**Parameter**	**Concentration (ng/mL)**			
	**LLOQ**	**LQC**	**MQC**	**HQC**
Mean of day 1	0.97	2.42	459.79	920.82
Mean of day 2	0.93	2.56	496.46	904.60
Mean of day 3	1.03	2.39	493.96	889.51
Average	0.98	2.46	483.41	904.97
SD	0.05	0.09	20.49	15.66
Precision (%CV)	5.33	3.54	4.24	1.73
% Accuracy	99.77	98.21	96.68	100.55
**Deoxyandrographolide (AP6)**				
**Parameter**	**Concentration (ng/mL)**			
	**LLOQ**	**LQC**	**MQC**	**HQC**
Mean of day 1	0.93	2.46	483.46	912.96
Mean of day 2	1.04	2.56	500.18	917.24
Mean of day 3	0.94	2.48	491.25	908.40
Average	0.97	2.50	491.63	912.86
SD	0.06	0.05	8.36	4.42
Precision (%CV)	6.22	2.10	1.70	0.48
% Accuracy	99.24	99.89	98.33	101.43

Table S3: Linearity of andrographolide (AP1), 14-deoxy-11,12-didehydroandrographolide (AP3), neoandrographolide (AP4), and deoxyandrographolide, (AP6) in plasma

Parameter	Back-calculated concentration (ng/mL)											
	CC1	CC2	CC3	CC4	CC5	CC6	CC7	CC8	CC9	CC10	CC11	CC12
**Andrographolide (AP1)**												
% Accuracy 1	83.25	90.62	105.52	88.55	90.29	113.42	105.99	103.24	96.37	92.42	96.55	102.01
% Accuracy 2	87.65	108.95	89.50	89.83	97.36	110.87	112.19	101.93	93.06	91.34	93.87	102.48
% Accuracy 3	85.61	99.84	89.50	103.95	103.12	102.76	104.71	97.99	88.06	94.48	93.12	102.09
Mean% Accuracy	85.50	99.81	94.84	94.11	96.93	109.02	107.63	101.05	92.50	92.75	94.51	102.19
SD	2.20	9.17	9.25	8.55	6.43	5.57	4.00	2.73	4.18	1.60	1.80	0.25
%CV	2.58	9.18	9.76	9.08	6.63	5.11	3.72	2.71	4.52	1.72	1.91	0.24
**14-deoxy-11,12-didehydroandrographolide (AP3)**												
% Accuracy 1	113.15	97.59	113.31	92.31	93.97	102.73	97.65	97.20	105.31	94.02	94.59	101.21
% Accuracy 2	110.39	94.72	106.88	103.67	94.09	100.85	100.72	91.73	104.10	99.06	93.35	100.10
% Accuracy 3	95.85	99.94	93.47	91.33	114.05	105.52	98.30	97.08	95.23	89.80	94.84	100.34
Mean% Accuracy	106.46	97.42	104.55	95.77	100.70	103.04	98.89	95.34	101.55	94.29	94.26	100.55
SD	9.30	2.62	10.12	6.86	11.56	2.35	1.62	3.13	5.51	4.64	0.80	0.58
%CV	8.73	2.69	9.68	7.16	11.48	2.28	1.64	3.28	5.42	4.92	0.85	0.58
**Neoandrographolide (AP4)**												
% Accuracy 1	88.58	95.03	87.14	85.91	91.16	98.00	97.20	100.73	108.06	99.01	94.94	99.75
% Accuracy 2	83.66	97.89	90.09	93.84	96.74	103.25	101.81	100.83	102.04	97.73	98.99	100.42
% Accuracy 3	100.56	107.57	105.81	103.83	90.42	97.07	103.96	100.44	92.50	89.81	97.90	102.94
Mean% Accuracy	90.93	100.16	94.35	94.53	92.77	99.44	100.99	100.67	100.86	95.52	97.28	101.04
SD	8.69	6.57	10.03	8.98	3.45	3.34	3.45	0.20	7.84	4.98	2.10	1.68
%CV	9.56	6.56	10.64	9.50	3.72	3.36	3.42	0.20	7.78	5.21	2.16	1.66
**Deoxyandrographolide (AP6)**												
% Accuracy 1	97.38	92.88	108.34	93.71	103.15	106.32	93.45	92.75	105.73	99.24	97.94	99.96
% Accuracy 2	113.87	90.98	114.20	88.18	91.64	110.39	105.32	101.54	98.53	96.28	94.56	100.96
% Accuracy 3	118.99	108.29	99.23	96.36	103.19	102.61	97.42	91.04	99.49	88.86	102.96	100.15
Mean% Accuracy	110.08	97.38	107.26	92.75	99.33	106.44	98.73	95.11	101.25	94.79	98.49	100.36
SD	11.29	9.49	7.55	4.17	6.66	3.90	6.04	5.63	3.91	5.35	4.23	0.53
%CV	10.26	9.75	7.04	4.50	6.70	3.66	6.12	5.92	3.86	5.64	4.29	0.53

Table S4: Linearity of andrographolide (AP1), 14-deoxy-11,12-didehydroandrographolide (AP3), neoandrographolide (AP4), and deoxyandrographolide, (AP6) in urine

Parameter	Back-calculated concentration (ng/mL)											
	CC1	CC2	CC3	CC4	CC5	CC6	CC7	CC8	CC9	CC10	CC11	CC12
**Andrographolide (AP1)**												
% Accuracy 1	92.47	86.73	88.37	88.85	87.05	104.52	89.65	93.69	103.67	100.90	100.33	99.50
% Accuracy 2	97.89	113.56	90.78	91.24	90.45	92.99	100.81	93.47	98.06	91.44	93.09	106.32
% Accuracy 3	83.05	99.48	96.92	102.54	97.51	105.46	87.15	98.05	93.43	94.04	97.87	103.21
Mean% Accuracy	91.14	99.93	92.02	94.21	91.67	100.99	92.54	95.07	98.39	95.46	97.10	103.01
SD	7.51	13.42	4.41	7.32	5.34	6.94	7.28	2.58	5.13	4.89	3.68	3.42
%CV	8.24	13.43	4.79	7.77	5.82	6.88	7.86	2.71	5.22	5.12	3.79	3.32
**14-deoxy-11,12-didehydroandrographolide (AP3)**												
% Accuracy 1	113.97	112.69	113.51	95.27	92.03	89.03	98.05	93.81	97.57	100.92	100.97	99.69
% Accuracy 2	109.16	100.15	112.10	103.80	88.64	92.54	93.33	97.41	91.31	93.63	95.93	104.85
% Accuracy 3	93.29	96.10	93.57	99.87	98.75	95.65	92.02	103.43	94.88	96.90	96.90	103.18
Mean% Accuracy	105.47	102.98	106.39	99.64	93.14	92.41	94.47	98.22	94.58	97.15	97.93	102.57
SD	10.82	8.65	11.13	4.27	5.14	3.31	3.17	4.86	3.14	3.65	2.67	2.63
%CV	10.26	8.40	10.46	4.28	5.52	3.59	3.36	4.95	3.32	3.76	2.73	2.57
**Neoandrographolide (AP4)**												
% Accuracy 1	93.70	98.10	99.69	91.03	107.99	95.06	97.20	97.17	98.76	109.12	99.07	98.75
% Accuracy 2	88.88	101.84	91.03	99.73	92.97	96.68	101.81	93.46	95.42	101.94	93.90	103.78
% Accuracy 3	88.58	109.26	106.37	96.40	113.49	106.70	103.96	88.04	90.31	105.19	95.29	102.31
Mean% Accuracy	90.39	103.07	99.03	95.72	104.82	99.48	100.99	92.89	94.83	105.42	96.09	101.61
SD	2.87	5.68	7.69	4.39	10.62	6.31	3.45	4.59	4.26	3.60	2.68	2.59
%CV	3.18	5.51	7.76	4.58	10.13	6.34	3.42	4.95	4.49	3.41	2.79	2.55
**Deoxyandrographolide (AP6)**												
% Accuracy 1	98.00	88.37	105.52	93.61	89.89	88.98	86.96	88.08	98.79	100.92	99.42	100.44
% Accuracy 2	117.97	96.92	112.31	94.07	104.40	94.87	94.00	93.20	92.31	94.10	97.14	103.93
% Accuracy 3	113.15	109.62	112.67	110.95	99.62	99.71	92.71	92.49	92.28	91.70	96.22	104.93
Mean% Accuracy	109.71	98.30	110.17	99.54	97.97	94.52	91.22	91.25	94.46	95.57	97.59	103.10
SD	10.42	10.69	4.02	9.88	7.39	5.37	3.75	2.77	3.75	4.78	1.65	2.36
%CV	9.50	10.88	3.65	9.93	7.55	5.68	4.11	3.04	3.97	5.00	1.69	2.29
